# Nanocellulose-Based Nanocomposites for Sustainable Applications: A Review

**DOI:** 10.3390/nano12193483

**Published:** 2022-10-05

**Authors:** Mohd Nurazzi Norizan, Siti Shazra Shazleen, Aisyah Humaira Alias, Fatimah Atiyah Sabaruddin, Muhammad Rizal Muhammad Asyraf, Edi Syams Zainudin, Norli Abdullah, Mohd Saiful Samsudin, Siti Hasnah Kamarudin, Mohd Nor Faiz Norrrahim

**Affiliations:** 1 Bioresource Technology Division, School of Industrial Technology, Universiti Sains Malaysia, Penang 11800, Malaysia; 2 Green Biopolymer, Coatings & Packaging Cluster, School of Industrial Technology, Universiti Sains Malaysia, Penang 11800, Malaysia; 3 Institute of Tropical Forestry and Forest Products (INTROP), Universiti Putra Malaysia, Serdang 43400, Selangor, Malaysia; 4 Advanced Engineering Materials and Composites Research Centre (AEMC), Department of Mechanical and Manufacturing Engineering, Universiti Putra Malaysia (UPM), Serdang 43400, Selangor, Malaysia; 5 Faculty of Biotechnology and Biomolecular Sciences, Universiti Putra Malaysia, Serdang 43400, Selangor, Malaysia; 6 Engineering Design Research Group (EDRG), School of Mechanical Engineering, Faculty of Engineering, Universiti Teknologi Malaysia, Johor Bahru 81310, Johor, Malaysia; 7 Centre for Advanced Composite Materials (CACM), Universiti Teknologi Malaysia, Johor Bahru 81310, Johor, Malaysia; 8 Centre for Defence Foundation Studies, Universiti Pertahanan Nasional Malaysia (UPNM), Kem Perdana Sungai Besi, Kuala Lumpur 57000, Malaysia; 9 Environmental Technology Division, School of Industrial Technology, Universiti Sains Malaysia, Penang 11800, Malaysia; 10 Department of Ecotechnology, School of Industrial Technology, Faculty of Applied Science, UiTM Shah Alam, Shah Alam 40450, Selangor, Malaysia; 11 Research Centre for Chemical Defence, Universiti Pertahanan Nasional Malaysia (UPNM), Kem Perdana Sungai Besi, Kuala Lumpur 57000, Malaysia

**Keywords:** biomass, cellulose, CNF, nanocellulose, nanocomposite, surface modification

## Abstract

Nanocellulose has emerged in recent years as one of the most notable green materials available due to its numerous appealing factors, including its non-toxic nature, biodegradability, high aspect ratio, superior mechanical capabilities, remarkable optical properties, anisotropic shape, high mechanical strength, excellent biocompatibility and tailorable surface chemistry. It is proving to be a promising material in a range of applications pertinent to the material engineering to biomedical applications. In this review, recent advances in the preparation, modification, and emerging application of nanocellulose, especially cellulose nanocrystals (CNCs), are described and discussed based on the analysis of the latest investigations. This review presents an overview of general concepts in nanocellulose-based nanocomposites for sustainable applications. Beginning with a brief introduction of cellulose, nanocellulose sources, structural characteristics and the extraction process for those new to the area, we go on to more in-depth content. Following that, the research on techniques used to modify the surface properties of nanocellulose by functionalizing surface hydroxyl groups to impart desirable hydrophilic–hydrophobic balance, as well as their characteristics and functionalization strategies, were explained. The usage of nanocellulose in nanocomposites in versatile fields, as well as novel and foreseen markets of nanocellulose products, are also discussed. Finally, the difficulties, challenges and prospects of materials based on nanocellulose are then discussed in the last section for readers searching for future high-end eco-friendly functional materials.

## 1. Introduction

For several decades, non-biodegradable plastics derived from petroleum-based materials have been widely used in many applications, particularly food packaging, due to their low cost, interesting technological features, and excellent mechanical and physical performances. The statistics show that the world’s population is expected to reach 9 billion by 2050, which directly increases the demand for plastics for humankind’s purposes. Thus, it will undoubtedly increase plastic waste, which contributes directly to global warming, water pollution, and air pollution [1,2]. Extensive research has been carried out to develop a substitute packaging material to minimize the environmental impact of petroleum-based packaging materials. Therefore, biomaterials are formulated into biocomposite materials to create bioplastics comparable to synthetic plastic to catch up with synthetic plastic. The global green revolution, which aims to reduce the negative ecological impact of polymeric materials, has resulted in research and development of various sustainable and renewable eco-friendly materials to replace petroleum-based materials [3,4,5].

Moreover, the critical importance of developing a new narrative of socioeconomic transformation to ensure the success of the United Nations’ Sustainable Development Goal (SDG) by 2030 has established a clear link between sustainable plant forest use and protection and vital ecosystem services. Due to this, researchers are currently focusing on renewable resources that are biodegradable and sustainable. Natural fiber is regarded as one of the most valuable types of renewable resources. Natural fiber is often used as a filler to strengthen bioplastic’s mechanical and barrier properties. The mixture of these materials resulted in promising biocomposite products that are safe, biodegradable, and long-lasting [6].

The idea of using natural fibers as an improvement in composites is not complete because of their outstanding fiber properties only, but also because of its impact on the profiteering of plant waste, forestry preservation, biodiversity, and the availability of fiber in abundance at a low cost. Natural fibers can also be obtained from agricultural and forestry byproducts such as rice straws, corn, pineapples, oil palm empty fruit bunch, Miscanthus × giganteus and sugar palm fibers [7,8,9]. Furthermore, a more distinct feature of fiber known as nanocellulose can be extracted using a chemical acid hydrolysis process. Due to its lightweight, high surface area, high mechanical strength, high aspect ratio, and low-density properties, nanocellulose is another promising material for use as a reinforcement filler in composite materials [10]. For example, Barbash et al. [11] had produced nanocellulose from Miscanthus × giganteus with transparency up to 82%, high crystallinity up to 76.5% and tensile strength up to 195 MPa. The findings show that the application of nanocellulose from Miscanthus × giganteus allows replacing synthetic reinforcing materials and more expensive sulfate unbleached pulp with wastepaper in the production of paper and cardboard. Nanomaterials made from cellulose have gotten much attention recently because they are renewable and long-lasting. Low toxicity and density, better tensile strength, and a high aspect ratio are just a few others of the unique qualities of nanocelluose. As cellulose nanocrystals (CNC) or cellulose nanofiber (CNF), nanocellulose is extracted from various biosources like wood, corn residues, cotton, sugar palm, bamboo, and rice straw [12,13]. The extraction and characterization of nanocellulose have been the focus of many researchers so far [14].

Acid hydrolysis, enzymatic hydrolysis, subcritical water hydrolysis, and mechanical processing have all been proposed to extract CNC from cellulose. The maximum yield of CNC recovered from cellulose is 70% to 80%, corresponding to a total yield of 20% to 30% from the original bioresource [15]. CNF are composed primarily of cellulose fibrils embedded in a learning matrix; thus, these CNF may have superior rigidity, tensile, and flexural properties [16]. As a result, an innovative approach using CNF can be a valuable tool for developing greener materials with improved properties and for the qualitative environmental management of sustainable materials. Nanocellulose derived from plant fibers is a potentially viable reinforcement to polymers for improving the properties of polymers in nanocomposite materials, and it is in high demand for various industrial applications. An adequate system of CNF for viable industrial applications may include qualitative and quantitative product performance in its entire life cycle. Due to their distinctive nano morphology, high crystallinity, excellent mechanical properties, and high specific surface area, nanocellulose, a new family of natural-based materials, have drawn increasing attention from academic research and industrial application with the development of nanotechnology [17]. Referring to Abe et al. [18], the obtained nonwoven nanostructures of CNF with a uniform width of 15 nm through a very simple mechanical treatment associated with interconnected pores and a very large surface-to-volume ratio denote the potential utilization of the CNF sheets for membranes, tissue scaffolds, filters and many other applications.

Furthermore, developing nanocellulosic materials will improve the end-user experience while allowing for more efficient manufacturing systems. A methodical engineering design process is used to create functional products. In general, as a whole, the engineering design process is critical in the development of effective manufacturing processes and technology for innovative products [19]. Prior to marketing, the use of the design process for the isolation of CNF will ensure product quality and sustainability requirements such as level of safety, ergonomics, size, height, thickness, and stress levels, as well as its quantitative life cycle assessment and cost. The primary role of a design process is to define the possibilities, limitations and suitability of CNF in the development of sustainable applications [20]. Given that the supply chain for nanocellulose is virtually limitless, this could be a golden opportunity to turn these agricultural wastes into wealth. Nanocellulose, in particular, has been used as reinforcement with positive results in a variety of sustainable industries, including medical, pharmaceutical, packaging, environmental, energy, and electronic applications [21,22,23,24,25,26,27,28]. The elastic modulus and tensile strength of nanocomposite films reinforced with CNCs showed a progressive rise with their addition. Several authors have reported the creation of eco-friendly and biobased nanocomposites in the field of food packaging [29,30,31,32]. As a whole, nanocellulose’s usage as reinforcement is already widely known. Particularly the mechanical and thermal properties are improved by microfibrils, nanofibrils or nanofibers, and cellulose nanocrystals.

Recently, nanocellulose-based nanocomposites are under extensive exploration and gained popularity among researchers [33]. There are several studies were reported on the potential application of nanocellulose-based nanocomposites. Mahfoudi et al. [34] and Norrrahim et al. [35] discovered the use of nanocellulose-based composite as an adsorbent for chemical contaminants remediation. They found that nanocellulose had better capability to adsorb chemical contaminants as compared to currently available adsorbents such as activated carbon, silica, zeolite, and chitosan. Besides that, Ferrer et al. [36] discovered on the recent developments in nanocellulose-based materials and their applications in packaging. They found that packaging materials developed from nanocellulose had better oxygen and water vapor barrier characteristics. Authors also reported that nanocellulose can sustainably enhance oxygen and water vapor barrier properties of the packaging materials when used as a coating, fillers in composites, and as self-standing thin films.

Despite the extraordinary properties of this composite, addressing an array of its potential applications in some new areas of interest such as automotive, aerospace, sensors and energy is still far from reaching to meet the industrial demands in a cost-effective way. Moreover, lack of studies were focused on the future prospects and challenges related to this area. 

In light of the above discussion, this review aims to provide an overview on recent advances of nanocellulose, including its sources, structure, isolation, modification of properties, processability and functionalization of nanocellulose. In addition, the description of the nanocellulose-based nanocomposites processing, including prospects and challenges of the potential applications, especially applicability towards sustainable applications, are also described and discussed, as well as the main properties of the nanoscale cellulose. This comprehensive review is expected to pave the way for innovative approaches to the creation of novel NC-based materials for a wide range of cutting-edge applications. 

## 2. Nanocellulose from Plant Fiber

**Figure 1:**
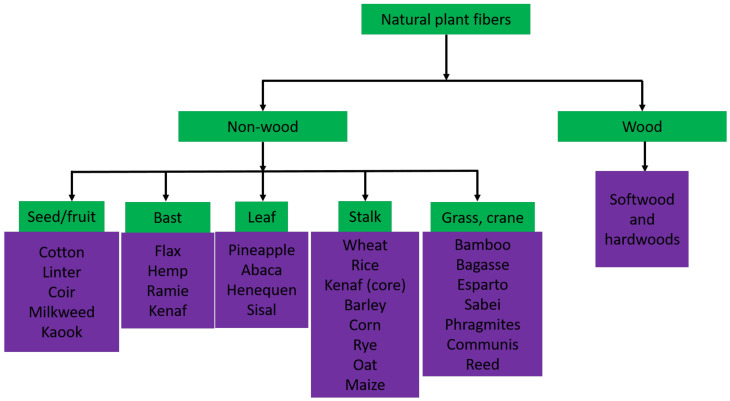
Classification of plant fibers based on their source. Reproduced from ref. [41].

Cellulose is known to be the most abundant, natural, renewable, and biodegradable polymer on earth. Cellulose can be found and produced in most plants, algae, animals, fungi, or bacteria [37]. Among them, plants and wood are the primary cellulose resources for natural biocomposites comprising microfibril cellulose, pectin, lignin, and hemicellulose [38,39]. Plant fibers have been applied since the industrialization era. Humans have utilized it for over 40,000 years for its advantages [40]. Apart from being the most dominant biopolymer on the planet, it also offers a variety of exciting characteristics, for example, excellent biocompatibility, lower density, substantial strength, and most beneficial mechanical properties at a lower cost. Plant fibers can be defined as cellulose-based fiber. The fibers are graded according to the position in the plant, for example, stalk, stem, bast, leaf, or seed of the plant, and these plant fibers then can be categorized as non-wood and wood plant fibers, as shown in Figure 1.

Since 150 BCE, cellulose has been utilized by the Chinese in the production of paper extracted from trees and plants. Cellulose researchers then found that cellulose in plants is formed from millimeter-sized filaments that contain microfibrils of nanometer measurements, which represent the fundamental structure of cellulose in all sources. Later in the 1980s, nanocellulose was known as the most regular plant-based polymer ever utilized isolated from its core material, cellulose [39]. Nanocellulose is one of the extended cellulose forms isolated as nanoparticles which is considered a useful class of futuristic materials. Nanocellulose can be divided into two major classes via, (1) nanostructured materials (cellulose microcrystals and cellulose microfibrils) and (2) CNF, CNC and bacterial cellulose (BC)) depending on their morphologies, origin and isolation techniques [42,43,44,45]. Klemm et al. [46] summarized the physical properties of this nanocellulose as listed in Table 1. 

Plants remain the main source of cellulose production, attributed to their abundance and low cost. Generally, all plants are eligible for the extraction of nanocellulose, including hardwood (e.g., eucalyptus, aspen, balsa, oak, elm, maple, birch, rubberwood) [42,47], softwood (e.g., pine, spruce, juniper, hemlock, yew, larch, cedar) [42], natural fibers (cotton, jute, bamboo, kenaf [48,49], roselle [50]), industrial and agricultural waste (e.g., corn husk, sugarcane bagasse, rice husk, sawdust, eucalyptus pulp, oil palm empty fruit bunch, potato peels, grasses) [51,52,53,54], and algae [55,56,57,58,59,60,61]. Among all these sources, agro-industrial biomass has shown a remarkably high potential to be extracted as nanocellulose. This is attributed to its abundant availability, which might help to give high productivity of nanocellulose on a large scale.

Nanocellulose extracted from plant fibers can be varied as well as their chemical composition. These properties have been reportedly associated with their type, origin, and age. The important constituent of the plant fibers is cellulose, hemicellulose, and lignin [40]. Structurally, plant cell walls are cytoplasmic matrices made of secondary walls that are enclosed within the primary walls forming an integral part of the plant structure. Cellulose, as the main constituent in plant fiber, is predominantly located in the secondary wall of plant cells [62]. It is confined to a cell wall made up of linear homopolysaccharides of β-1, 4-linked anhydro-D-glucose unit of cellobiose repeating unit. Each repeating unit consists of three hydroxyl groups which lead to the formation of strong inter-and intra-hydrogen bonds with an end-to-end glucose unit that imparted strongly packed crystalline cellulose fiber. This formation however is dependent on the origin of the fibers and the extraction method. Hemicellulose is another major component of the plant fibers composition. It helps to bond the cellulose and lignin and forms a link between hydrophobic lignin and hydrophilic cellulose, imparting rigidity to the plant. Lignin on the other hand is a phenolic polymer acting as a protective barrier wrapping the cellulose and hemicellulose [40]. Meanwhile, it is reported that the plant fibers possess a diameter of approximately 13–22 µm, whilst their crystallinity is in the vicinity of 44–65% before being extracted to nanocellulose. These fibers are then isolated through chemical, biochemical, or mechanical techniques to produce nanocellulose with one dimension less than 100 nm [38]. In most cases, CNF possesses a diameter in the vicinity of 4–20 nm, *L/D* > 100, where the production generally indicates the fibrillation of the microfibrils from the cell wall of the plant fibers. Meanwhile, the physical dimension of CNC, designated by length (*L*), diameter (*D*), and aspect ratio (*L/D*) rely mostly on the cellulosic source and method of production. The CNC from cotton was reported to be shorter with an aspect ratio of 10–30, whilst the dimension of CNC from wood was reported with a length of 100–200 nm with a diameter of 3–5 nm [63]. The dimensions of nanocellulose produced from different plant fibers were listed in Table 2. In addition, Table 3 shows the nanocellulose production yield obtained per 100 kg of raw material from various production reports in the literature.

Regardless of their category, nanocellulose particularly originated from plant fibers has gained attention from research and industries. With excellent properties and biodegradability, nanocellulose-based plant fibers have been utilized in many fields including nanocomposites, surface-modified materials, and transparent paper with special functions [87].

## 3. Isolation of Nanocellulose from Plant Fiber

Nanocellulose extracted from plant fibers are also known as cellulose-based nanomaterials isolated from plant fibers with at least one dimension in the range of nanometers. These materials, regardless of their category, have been utilized as an effective material in wider applications based on their economical, eco-friendly, biodegradable, and other remarkable properties. The production of nanocellulose, whether it is CNC or CNF, can be extracted or isolated from plant fibers via a top-down approach [88,89]. This approach involves various techniques, including chemical technique, mechanical technique, enzymatic technique, and physicochemical techniques. The implementation of these techniques needs to be determined based on the parameters needed for the nanocellulose, for example, the porosity condition, crystallinity properties, surface area, and the chain length of the nanocellulose [90].

The preparation of nanocellulose involves two major steps including (i) pre-treatment, which includes the cleaning of the fiber, removal of the extractives, pulping or alkaline treatment, and (ii) the isolation of the nanocellulose involving the depolymerization and mechanical isolation. Commonly, the CNC is isolated via an acid hydrolysis process whilst the CNF is extracted through various steps of mechanical treatments including grinding, cryo crushing, steam explosion, microfluidation, superheated steam, ultrasound, homogenization, etc. [91,92]. Despite different stages of the isolation process, the effectiveness of the nanocellulose production depends on the efficiency of the non-cellulosic removal during the pre-treatment stages as this process allows the intermolecular interactions that become the main drivers of its behavior and final properties [93]. The extraction of the nanocellulose may follow the steps listed below:

I. Dewaxing of the fiber;
II. Mercerization or alkalization;
III. Bleaching;
IV. Depolymerization (chemical, mechanical or biological methods);
V. Isolation.

**Figure 2:**
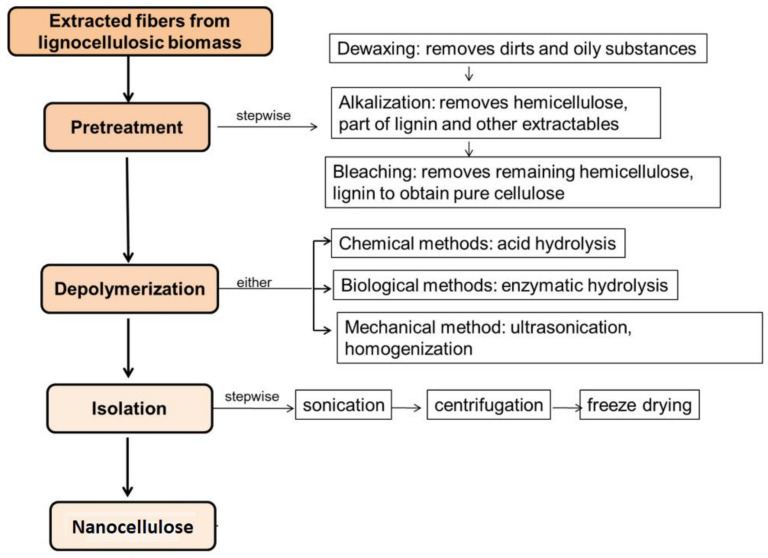
The schematic showing the general steps for nanocellulose preparation. Reproduced from [51].

Nevertheless, these steps greatly depended on the types of fibers used. For example, cotton fiber might not require intense cleaning considering its low content of extractives like wax, oil, and pectin. Whilst other fibers like sisal and pineapple leaves will require all the steps. Therefore, it is important to study the compositional properties of one fiber before the extraction process. The pictorial overview of the steps involved in the preparation was shown in Figure 2.

### 3.1. Dewaxing

Dewaxing is the initial step taken for the preparation of nanocellulose. This process involves the removal of the minor components referred to as extractives. These extractives include the components in fiber such as wax, phenolics, pigments, and oil. The dewaxing process is performed using a mixture of organic solvents with varying polarities, for example, benzene-methanol, hexane-ethanol, ethanol-toluene, and benzene-toluene. The process was also conducted at elevated temperatures to improve the efficiency of the process. For example, Morán et al. [94] have carried out dewaxing of sisal fibers using the mixture of toluene-ethanol at the ratio of 2:1 *v*/*v* in Soxhlet for 6 h. A similar dewaxing condition was also applied by Almendárez Camarillo et al. [95], where the dewaxing process was carried out upon pineapple leaves fiber for the preparation of nanocellulose extraction using a mixture of toluene-ethanol with the ratio of 1:2 *v*/*v* for 6 h.

**Figure 3:**
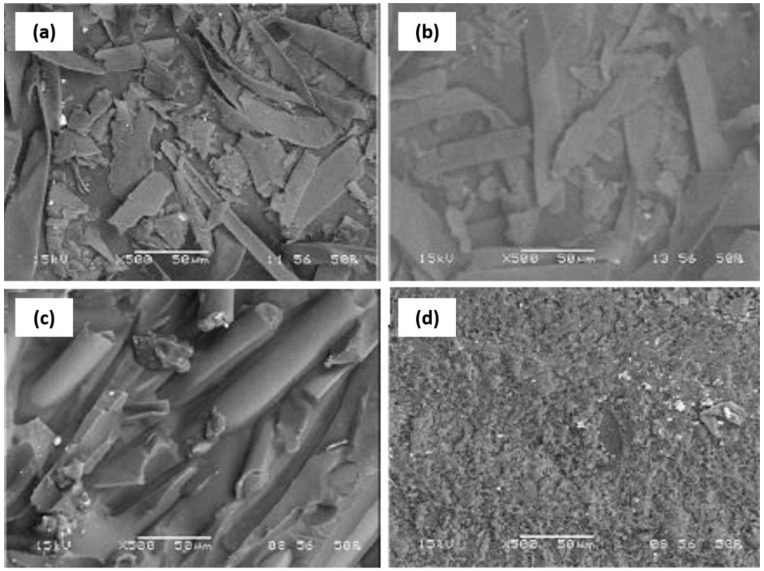
(**a**) Raw kapok fiber, (**b**) dewaxed kapok fiber, (**c**) raw kapok fiber reinforced epoxy composites and (**d**) dewaxed kapok fiber reinforced epoxy composite. Reproduced from [97].

A similar mixture was also applied for dewaxing Kapok fiber with an aim to remove the wax and impurities from the fiber surface. Pai et al. [96] reported that the dewaxed Kapok fiber showed higher crystallinity compared to raw kapok fiber from 54 to 63%, attributed to the regular arrangement attained by removing the wax and impurities using a mixture of alcohol and benzene at the temperature of 65 °C for 2 h. This property was proved by the surface morphology depicted in Figure 3 of raw and dewaxed kapok fiber. Dewaxed kapok fiber showed better uniformity and homogeneity when incorporated in epoxy composite compared to that raw kapok fiber. Meanwhile, the dewaxing process is often followed by the treatment using a very diluted alkaline solution (1 wt.%) at elevated temperature, which is also known as the pre-alkalization process. This process assists in the swelling of the fiber for the eventual alkaline treatment [51]. Despite its advantages of assisting the isolation of nanocellulose, many findings show the number of studies that did not apply the dewaxing and pre-alkalization process may cause an additional cost to the research [51].

### 3.2. Alkalization or Mercerization

The production of nanocellulose needs to be pre-treated to remove the non-cellulosic components, including lignin and hemicellulose. Other than dewaxing, the alkaline pre-treatment and bleaching process was very important for the preparation of nanocellulose, whether it is CNF or CNC [98]. In general, alkaline treatment was conducted to break down the bundle of fibers into individual fibers. This process allows the increment aspect ratio of the fibers and makes the surface of the fiber rougher, which helps increase the interfacial properties of the fiber with the matrix in the composite component [99]. The alkaline route of delignification involves pre-treatment using alkalis like sodium hydroxide (NaOH), potassium hydroxide (KOH), calcium hydroxide (Ca(OH)_2_), ammonia (NH), and sodium carbonate (Na_2_CO_3_) [90]. The mechanism of delignification is the de-crystallization of the cellulosic materials, increment of the surface area, and reduces the degree of polymerization. Alkaline treatment is also referred to as the mercerization process, where in this process the cellulose component will interact with a relatively concentrated aqueous solution of a strong base such as NaOH. The Na^+^ cations will penetrate intercrystalline spaces, leading to swelling of the cellulose and breaking the intermolecular hydrogen bond [100]. The sufficient swelling allows the reduction of the linear structure of fiber, as well as the change in mechanical properties depending on the parameters set for the process, including time and concentration of the alkali [101].

The mechanical deficiency of the natural fibers lies in their hydrophilic nature, which influences the adhesion problems to a hydrophobic matrix. The alkaline treatment, on the other hand, imposed a rough fiber surface, which led to the enhancement of a larger surface area and improved interlocking properties with the matrix [99]. These properties established the strength and toughness of the composite system. For example, Nayan et al. [101] investigated the effect of mercerization on pineapple leaf fiber (PALF) using different concentrations of NaOH solution. Through the finding, the author found that the alkaline treatment had significantly influenced the physical and morphological properties of the PALF. The mercerized PALF showed rough surface topography with higher aspect ratio. In addition, mercerized PALF showed better orientation with compact spaced fiber resulting in higher exposed cellulose due to loss of cementing materials after the mercerization process. The improvement in tensile strength was also reported for the mercerized PALF at optimum concentration of NaOH. This process proved its suitability for the mechanical improvement of cellulosic materials.

Another important chemical pre-treatment of fiber is the pulping process. The pulping method has become the most important process, especially for the pulp and paper industries. This process aims to keep the structure of the wood fiber intact while enhancing the removal of lignin. The pulping method can be divided into three major categories viz. chemical, mechanical, and semi-chemical. The most common chemical pulping includes sulfate (kraft) and sulfite pulping. In general, during this process, the lignin must be removed to allow the separation of cellulose fiber and allow it to be dispersed in water and reformed into a web using sodium hydroxide [93,102]. The sulfate process or Kraft pulping was invented by a German chemist named Carl F. Dahl in 1879. This process was modified as cooking liquor using sodium sulfate instead of sodium hydroxide. The sulfate pulping process is also known to be much stronger and nowadays has become a well-known chemical wood pulping process using sodium hydroxide (NaOH) and sodium sulfite (Na_2_S). Currently, the Kraft pulping was carried out using NaOH and Na_2_S according to parameters of 2–4 h cooking time, at a temperature between 170–180 °C [93]. This process can produce a superior product in terms of strength; however, it needs a high sequence of bleaching to achieve the desired level of brightness.

However, the sulfite process used sulfur dioxide as the main component of the pulping liquor, which was found by Benjamin C. Tilghman [102]. The mechanism of this pulping process lies in the applications of SO_2_ obtained from hydrogensulfite (HNO_3_^−^) for the modification of lignin and the use of some cationic bases (Ca^2+^, Mg^2+^, Na^+^, NH_3_^+^) to avoid chromophore formation from the residual lignin on the fibers, which might lead to the hydrolysis process. In general, the standard operating temperatures range from 130 °C to 140 °C for 6–8 h at a pressure of 100 psi, and a pH up to 5 [93].

In addition, chemical pre-treatment also can be combined with mechanical methods where wood chips are partially softened or digested using chemicals, such as sodium hydroxide, sodium carbonate, or sodium sulfate. After the digesting process, the pulp was subjected to a disk refiner. This method is also able to achieve as high a yield as possible with the best possible strength and cleanliness [102]. Another well-known method for pulping is the organosolv pulping process. This method used organic solvents including methanol, ethanol, and acetic acid as cooking solvents for the pulping process. This method has been applied widely due to its efficiency to treat hard and soft wood, as well as non-wood materials.

### 3.3. Bleaching

Chemical pre-treatment such as mercerization and the pulping process provides the extracted cellulose fiber with mechanical strength. Nevertheless, there is still a lignin component present on the surface of the fiber. Therefore, the bleaching process is commonly applied to remove the residual lignin without impacting the fibers mechanical properties [93]. Bleaching through chemical techniques can lead to a brighter color of the pulp, indicating the removal of the lignin.

The bleaching process can be carried out using oxidizing agents including chlorine dioxide (ClO_2_), oxygen (O_2_), ozone (O_3_), and hydrogen peroxide (H_2_O_2_). During the bleaching process, fibers will interact with the oxidizing agents, followed by the sequence of alkaline washing that generates changes to the surface as these processes aim to modify the existing lignin while increasing its solubility [93].

There are also reports regarding the effect of bleaching the chemical pulps using chemicals like chlorine, ClO_2_, and H_2_O_2_. These chlorinated by-products as the production of higher ClO_2_ substitution leads to the release of harmful chlorinated organic compounds. Meanwhile, the application of hypochlorite, which is commonly used for bleaching, also caused the generation of chloroform as a by-product and is strictly regulated by government agencies in many countries. Therefore, due to high market demands for chlorine-free products, the industry had changed its implementation to a elementary chlorine-free (EFC) and total chlorine-free (TCF) bleaching process, by substituting oxygen-based chemicals with hypochlorite, Cl_2_, and ClO_2_.

### 3.4. Depolymerization

#### 3.4.1. Chemical Techniques

Chemical routes for nanocellulose isolation can be divided into several types, such as acid pre-treatment, oxidation, pre-treatment using ionic solvent, and other types of solvent isolation techniques [90]. Chemical pre-treatments have offered efficient routes for the extraction of nanocellulose as they are able to enhance the reactivity of the hydroxyl group through the breakage of hydrogen bonds, thus increasing the exposed surface of the fibers [103].

The acid pretreatment for the nanocellulose extraction involves diluted and concentrated acids, for example sulfuric acid (H_2_SO_4_), hydrochloric acid (HCl), hydrogen phosphoric acid (H_3_PO_4_), nitric acid (HPO_3_), oxalic acid, maleic and some heteropoly-acid (HPAs). Acid hydrolysis is the main process for the extraction of nanocellulose particularly from plant fibers. With the combination of ordered and disordered regions of the cellulose chains, the disordered regions can be easily hydrolyzed by acid and the ordered parts are left remaining [104]. Acid pre-treatment usually works by effectively breaking the amorphous cellulose and extracting the nanocellulose in the form of CNC. Sulfuric acid is the most preferable acid used in the hydrolysis process by strongly isolating nanocrystalline cellulose and effectively dispersing as a stable colloid system due to esterification of the hydroxyl group by sulfate ions. The main factors controlling the process include reaction time, temperature, and acid concentration [104]. The application of the acid, on the other hand, imparts major drawbacks owing to the toxic and corrosive nature of the acid itself [105,106]. Another major drawback is the production of acid wastewater generated from the washing process to neutralize the pH value of the nanocellulose suspension [107]. Instead of neutralizing using water, an alkaline such as sodium hydroxide (NaOH) was used to neutralize the pH value. For example, Sabaruddin et al. [108] extracted CNC from kenaf core using 64% sulfuric acid. The neutralization of the CNC was washed out by washing with deionized water and centrifugation, followed by neutralization using a few drops of strong alkaline (NaOH) until the pH of the suspension was neutral.

In most cases, despite the types of the chemical used, whether it is acid or alkali, this pre-treatment may involve hydrolysis. For example, the application of acid in the pre-treatment process involves the cleavage of the inter- and intra-molecular bonds between hemicellulose and lignin, whilst hydronium ions function by separating cellulose from lignin and hemicellulose. Meanwhile, the alkali hydrolysis works by removing the hemicellulose through saponification of the inter-molecular ester bonds between lignin and hemicellulose, also breaking the bonds between cellulose, hemicellulose, and lignin [103].

Another important method used for the isolation of the nanocellulose is oxidation, which refers to ozonolytic treatment using hydrogen peroxide (H_2_O_2_), dioxygen (O_2_), and ozone (O_3_) to allow the delignification process [105]. The application of ozone has been considered the best agent for delignification as the H_2_O soluble properties mainly attack the conjugated C=C bonds and aromatic compounds. Among all, wet oxidation is the most applicable oxidation process based on its effectiveness to remove 50–60% of the lignin content from the cellulosic waste and continue in either air or oxygen in the presence of H_2_O at a pressure of 5–20 MPa and temperature in the vicinity of 150 °C to 350 °C [109,110,111].

TEMPO-oxidation is another common method that lies under the chemical pre-treatment process, performed under aqueous and mild conditions. The mechanism of this technique is the oxidation of the glucose through selective oxidation of C6 primary and converting it to the carboxylate group via the aldehydic group. The oxidation of cellulose using 2,2,6,6 tetramethyl-1-piperidinyloxy (TEMPO) using NaBr as a catalytic agent at pH 10–11 at room temperature. In addition, this method proved to be an effective route to isolate CNF through relatively low-energy processing by imposing a negatively charged group (carboxylate one) onto the cellulose surface. Repulsive energy generated by carboxylate groups led to the lesser aggregation of the processed fiber led to better stability and flow behavior of the nanocellulose suspension [103,112]. The CNF isolated through the TEMPO-oxidation process was reported by Phanthong et al. [104] to always have uniform width (3–4 nm) with a high aspect ratio, which is useful for transparent and flexible display, as a gas-barrier film for packaging, and as a nanofiller in composites materials. In addition, TEMPO-oxidation has been selected as one of the most economical processes for producing nanocellulose by saving energy by 100 to 200 times as compared to the consumption of energy of the cycle of a high-pressure homogenizer (700–1400 MJ/Kg), reducing it to less than 7 MJ/Kg. An interesting study was conducted by Saito et al. [113] on the preparation of CNF with crystallinities around 65–95% through the TEMPO-mediated oxidation method. The optimized parameters and results show that the TEMPO-mediated oxidation of native cellulose at pH 10 is optimum for shortening the oxidation time. The CNF obtained around 3–4 nm in width and a few microns in length makes it possible to provide almost transparent and highly viscous dispersions. Almost all the primary hydroxyl groups facing the outer side of each cellulose fibril (i.e., about one of every two glucose units of the cellulose chains on the cellulose fibril surface) needs to be oxidized to the carboxylate ones by the TEMPO-mediated oxidation in order to obtain mostly individualized CNF in water dispersions. They also found that the obtained carboxylated groups formed on CNF surfaces with a density of 3.4 groups/nm^2^ or 0.54 C/m^2^) lead to stable dispersions, and the results found that both never-dried and once-dried celluloses give similar CNF/water dispersion when the conditions in terms of pH, CNF width, length, and density are fulfilled. A CNF purified from wood from chips of *Cryptomeria japonica* and sea tunicate (*Halocynthia papillosa*) has been prepared by Saito et al. [114]. Both CNFs were fully dispersed in water via a topochemical modification using a TEMPO-mediated oxidation process. Referring to Saito et al., most of the wood nanofibrils sonicated for 5 min were at least 1 μm long, After 80 min of sonication, the length of the nanofibrils was dramatically reduced to an average of 278 nm, and most of the nanofibrils had fragmented to give rigid rod-like particles. Further sonication for up to 200 min, however, did not markedly reduce the nanofibril length; the measured average length of nanofibrils was still as high as 272 nm after 200 min of sonication. Only the longer nanofibrils, remaining after 80 min of sonication, were further fragmented under the application of an additional 120 min of sonication. As a consequence, the length distribution became somewhat narrower. The strength of individual nanofibrils was then analyzed through the Weibull function well-established for the description of the fracture statistics. The mean strength of the wood cellulose nanofibrils ranged from 1.6–3 GPa, depending on the method used to measure the nanofibril width. The highly crystalline, thick tunicate cellulose nanofibrils exhibited higher mean strength of 3−6 GPa.

In most cases, TEMPO-oxidation pre-treatment was followed by a mechanical disintegration treatment using a mixer, refining and homogenization, microfluidization, ball milling, grinding, cryocrushing, steam explosion, ultrasonication, intense stirring and centrifugation [103,115], etc. Other than the above techniques involving chemical routes, nanocellulose also has been reported to be extracted using an ionic solvent. The solvents used for the extraction pre-treatment using various types of the solvent including methanol (MeOH), ethanol (EtOH), butanol (C_4_H_9_OH), triethylene glycol (C_6_H_14_O_4_), tetrahydrofuran (C_4_H_8_O), ethers, ketones, benzene (C_6_H_6_), and so forth [105,116]. However, this technique was not preferred, attributed to the high cost of the organic solvents and instrumental setup. In addition, the application of the volatile organic solvent was restricted, thus limiting its application in the industry [117].

#### 3.4.2. Mechanical Technique

Mechanical routes for nanocellulose production are the processes where cellulose fibrils were subjected to high shear force to cleavage the cellulose fiber in the longitudinal axis, resulting in CNF. The most common mechanical approaches for CNF production include grinding, cryocrushing, high-pressure homogenization, ultrasonication, steam explosion, and ball milling methods [118].

High-pressure homogenization (HPH) is one of the popular techniques for the mechanical isolation of nanocellulose at both laboratory and industrial scales [91]. This method was preferred due to its simplicity, high efficiency, and no requirement for organic solvent for biomass refining [89]. The mechanism of this technique is bypassing the cellulose slurry into the vessel with high pressure and high velocity, which the impact force and shear force in the fluid lead to the cleavage of cellulose microfibrils into nanometer-size cellulose [118]. During this process, the suspension viscosity will be changed from low to high viscosity. Therefore, to avoid clogging during the homogenization process, pre-treatment of cellulose, for example, steam explosion, microfluidization processor, or other methods is needed [119]. Ultrasonication is a process of defibrillation of cellulose fiber through the hydrodynamic force of the ultrasound. This process was carried out via large intensity ultrasonic waves, at temperatures and pressure greater than 5000 C and 500 atm, which effectively fibrillate the nanocellulose fiber [105].

Ball milling is one of the most common mechanical techniques used for nanocellulose production. This process takes place in a milling rotating jar consisting of milling balls (or grinding media) of various sizes. This process allows the defibrillation of the cellulose fibers through the movement of the balls in the rotating jar and creates a shear force to defibrillate the cellulose fiber. The ball milling process was used to grind native cellulose fibers to produce amorphous cellulose with decreasing crystallinity, size, and morphology, with changes in the crystalline lattice [118]. Another method for the isolation of nanocellulose is cryocrushing, where the fiber is treated with liquid nitrogen to obtain ice crystals to impart larger pressure within the cell wall, leading to an explosion of the cell wall to form the CNF [105].

#### 3.4.3. Enzymatic Technique

**Figure 4:**
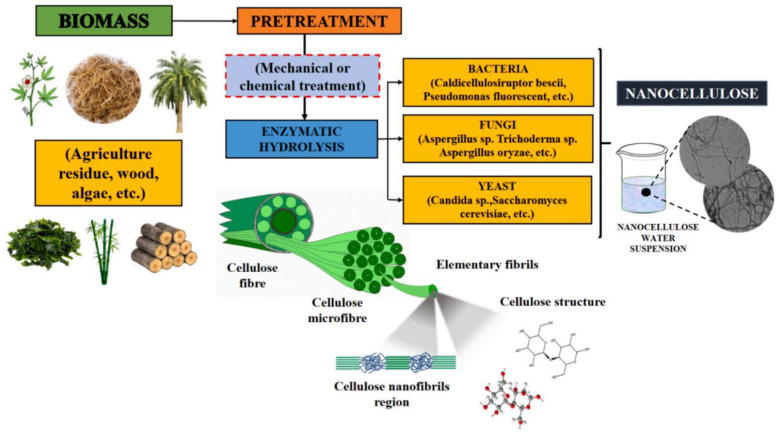
The extraction of nanocellulose via enzymatic hydrolysis using different types of microorganisms. Reproduced from [57].

The enzymatic hydrolysis process of producing nanocellulose is a promising eco-friendly method using a set of enzymes known as cellulase, which catalyze the breakdown of the carbohydrate cell wall and that assist the isolation process of nanocellulose [57,120]. The enzymatic hydrolysis can be performed using various types of microorganisms, including bacteria, yeast, and fungi, as depicted in Figure 4. The process involves a complex process that includes the removal of pectin and hemicellulose, as well as the utilization of enzymes to break down the cellulose segment by weakening and disrupting the fiber’s cell wall structure [57]. The complete hydrolysis process then requires the synergistic action of at least two of the three groups of cellulases known as endoglucanases, exoglucanases, and cellobiohydrolases. Among all, the production of nanocelluloses using endoglucanases showed the greatest interest due to their action on amorphous cellulose [121].

Alternatively, enzymatic hydrolysis has been utilized for acid hydrolysis as it helps to reduce the environmental impacts of the process, for example, lower water demand and the absence of chemical residues [122]. The application of enzymes in the production of nanocellulose was also reported to be coupled with chemical and mechanical treatment. The efficiency of this process also depends on many factors including crystallinity, average molecular weight, polymorphism, and lignin or hemicellulose contamination. For instance, Babicka et al. [121] have applied the enzymatic hydrolysis process using an enzyme from *Trichoderma reesei* for the production of nanocellulose with the combination of two types of ionic liquid: 1-ethyl-3-methylimidazole acetate (EmimOAc) and 1-allyl-3-methylimidazolium chloride (AmimCl) from microcrystalline cellulose (MCF). The application via a two-step process involving enzyme pre-treatment and hydrolysis using different ionic liquids showed a different range of particle sizes and structure properties. The hydrolysis of nanocellulose with EmimOAc imparted a larger particle size of 200 nm compared to the one obtained through AmimCl treatment with a particle size in a range of 30–40 nm. The basic cellulose I was obtained for nanocellulose treated with AmimCl, whilst cellulose II occurred for the cellulose treated with EmimOAc.

### 3.5. Isolation

Despite the efficiency of the method to extract the nanocellulose from the plant fibers, post-treatment such as purification from the excessive use of chemicals, or the agglomeration of the nanocellulose after the extraction process, require the after treatment to produce stable nanocellulose. Further, post-treatment includes solvent elimination, neutralization, washing, purification, filtration, centrifugation, sonication, dialysis, fractionation, surface modification, stabilization, and drying (freeze-drying or spray-drying) [123].

Acid hydrolysis is a common method to produce CNC using a strong acid, sulphuric acid, with a concentration in the range of 40–65%. However, the presence of sulfate ester at the cellulosic surface imparted low thermal stability but permitted well-dispersed individual CNC bundles in aqueous media [48,108]. Sabaruddin et al. [108] applied the post-alkaline treatment toward produced CNC to reduce the effect of dehydration reaction and thermal instability. The CNC suspension was neutralized using strong bases of NaOH and showed improvement in the thermal properties by shifting the melting temperature to a higher value. Other than the use of alkaline post-treatment, the presence of the acid in the cellulose surface can be reduced via washing, repeating the sequence of centrifugation, dialysis, and filtration. However, these methods led to excessive use of water. Nevertheless, the influence of the techniques is very important to confirm the purity of the nanocellulose.

The nanocellulose is commonly produced in the form of suspension at the end of the fibrillation process. The production of the nanocellulose is usually followed by an ultrasonication treatment in order to disintegrate the aggregates of the nanocellulose particles. Different sonication techniques have been applied in the production of nanocellulose. As reported by Csiszar et al. [124], the applied ultrasonication did affect the surface charges of the cellulose crystallites. The ultrasonication is usually carried out on the nanocellulose suspension prior to film casting to increase the pitch of the chiral nematic phase in suspension and move the reflection band of the final iridescent film to a longer wavelength. This study was also conducted by Peng and Via [125] towards CNC suspension to investigate the effect of ultrasonication or homogenization on the casted film property. The ultrasonication was reported to have significantly reduced the suspension viscosity with the highest degree of crystallinity.

The physical properties of the nanocellulose at the end of the process also play a major role in its applications. As nanocellulose was produced in the form of aqueous slurry, this has limited some of its end applications, for example, in the manufacture of plastic composites. Therefore, the requirement of the drying process was seen as very crucial. The desirable good drying method will affect the nanocellulose morphology and its properties [126]. Producing the dry form of nanocellulose can be achieved through several routes, including freeze-drying process, spray drying process, supercritical drying, or water evaporation. Peng et al. [126] studied the effect of different drying methods on nanocellulose. Both CNC and CNF were subjected to four separate drying methods: air-drying, freeze-drying, spray-drying, and supercritical-drying. The study found that air-drying and spray-drying of CNF imparted more thermal stable properties, whilst CNC dried via air-drying, freeze-drying, and spray-drying showed similar onset temperature for thermal degradation. The crystallinity indices of nanocellulose were then different for each drying method. The CNF is pure cellulose I whilst the dried CNC consists of cellulose I and II. The content of cellulose II in CNC, on the other hand, changes with each drying method. At the end of this study, it is concluded that spray-dried nanocellulose is recommended especially for the application with non-polar thermoplastics due to its higher thermal stability and crystallinity index.

A study for the preparation of CNF without any chemical modifications has been successfully conducted by Kondo et al. [127]. In this study, equivalent aqueous suspensions of cellulose are ejected from dual nozzles and collide at high speed and pressure ranging from 70 to 270 MPa. They found that the ACC method is a rapid means of processing cellulose into nanofibers using only water and without chemical modification and is applicable not only to cellulose but also to other polymeric materials having hierarchical structures.

## 4. Modification of Properties, Processability and Functionalization of Nanocellulose

Nanocellulose’s strong intermolecular hydrogen bonding interaction and high surface energy caused it to aggregate in a variety of environments, according to research. Therefore, nanocellulose’s surface characteristics and interfacial compatibility are critical to its functionalization and practical application [61,128,129,130]. There are several reviews on the surface modification of nanocellulose to improve interfacial compatibility that have been published so far [131,132]. The nanocellulose fiber has two major disadvantages: (1) a large number of hydroxyl groups, which causes the product’s structure to be gel-like, and (2) high hydrophilicity, which limits its applications. Thus, the goal of the modification is to improve these two drawbacks in order to improve the characteristics of nanocellulose and expand the range of natural fiber uses [133]. For reproducible research results and for all envisioned nanocellulose products to meet quality control criteria, stable dispersion is required. Due to the strong contact between the surface hydroxyls and the solvent molecules, nanocellulose can disperse in some strong polar solvents (particularly water). However, at the micro level, hydrogen bonding between nanofibers causes aggregation. Furthermore, nanocellulose’s hydrophilicity makes it difficult to disperse in hydrophobic media such as low polar and nonpolar chemical solvents, as well as most polymer matrices used in composite preparation.

**Figure 5:**
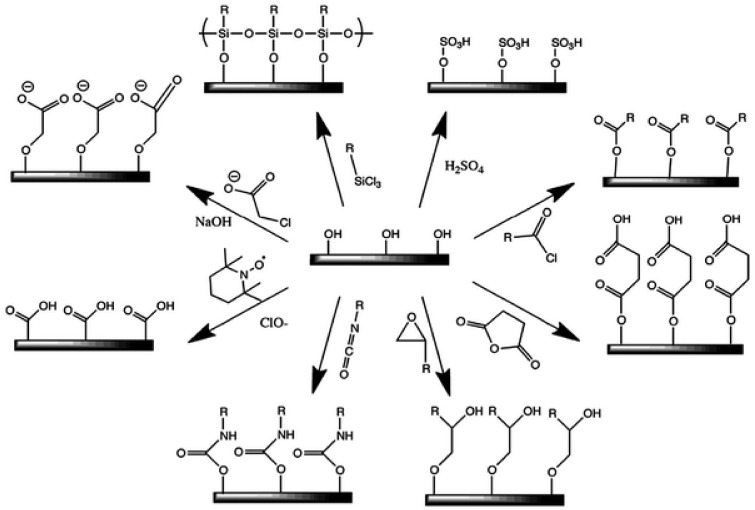
Several common types of surface modifications of nanocellulose: (clockwise from top-right) sulfuric acid treatment provides sulfate esters, carboxylic acid halides create ester linkages, acid anhydrides create ester linkages, epoxides create ether linkages, isocyanates create urethane linkages, TEMPO mediated hypochlorite oxidation creates carboxylic acids, halogenated acetic acids create carboxymethyl surfaces, and chlorosilanes create an oligomeric silylated layer. Reproduced with permission from Ref. [137].

Until now, establishing effective nanocellulose dispersion in various mediums has posed a significant difficulty in the development of marketable cellulose nanomaterials. Better surface modification depending on the properties of specific media, with the goal of changing the surface hydrophilicity and improving compatibility, is an effective way to improve nanocellulose dispersity. In order to modify nanocellulose, several methods such as silane grafting, acetylation, alkylation, esterification, 2,2,6,6-tetramethylpiperidin-1-oxyl (TEMPO) oxidation, carboxymethylation, and the use of nanoparticles were used [28,60,134,135,136]. Recent advances have concentrated on the surface functionalization of nanostructured cellulose to maximize its compatibility and properties with various sources. Recently, scientists have used a variety of modification techniques to alter the structure and surface of nanocellulosic materials as presented in Figure 5.

**Figure 6:**
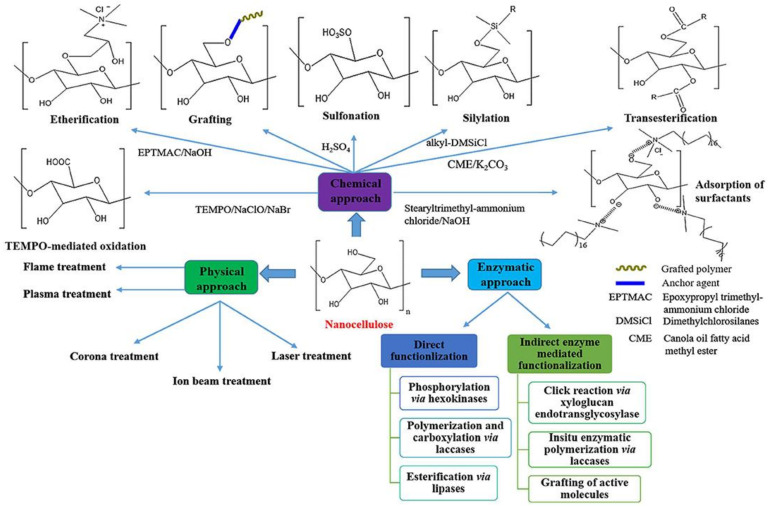
Schematic diagram of the most frequently used methods of functionalization of nanocellulose. Adapted from Ref. [138].

The discovery of surface hydrophobization and functionalization is required to broaden the application of nanocellulose since self-agglomeration and hydrophilicity caused by OH groups that are naturally present on its surface are still its main drawbacks. Figure 6 demonstrates a schematic diagram of the most commonly used method of functionalized nanocellulose. Numerous surface functionalization options for nanocellulose include chemical approach, physical approach and enzymatic approach.

### 4.1. Physical Approach of Functionalization of Nanocellulose

Numerous surface treatment techniques have evolved over recent years with the primary goals of lowering the surface energy of nanocellulose and increasing their degree of dispersion in a variety of solvents. Table 4 presents the common physical approach used in functionalization of nanocellulose. Plasma treatment, flame treatment, corona treatment, laser treatment and ion beam treatment are a few examples of physical surface functionalization procedures.

### 4.2. Chemical Approach of Functionalization of Nanocellulose

According to Islam et al. [148], it has been observed that adding non-modified biofiber to a polymer matrix dramatically reduces the tensile strength and ductility of green composites due to weak fiber–polymer interfacial adhesion. However, this may increase stiffness and thermomechanical properties. It is clear from this that in order to customize the features of this novel class of nanocomposites, composite materials must be modified or a compatibilizer must be used. Numerous treatments are possible due to the availability of hydroxyl groups on the surfaces of lignocellulosic fibers and/or nanofibers. The various alterations made to nanocellulose materials by chemical approach are listed in Table 5 along with any potential impacts.

### 4.3. Enzymatic Approach of Functionalization of Nanocellulose

The usage of enzymes in the synthesis of nanocellulose is the enzymatic approach to the functionalization of nanocellulose that has been conducted by several researchers. Michelin et al. [158] study had a particular focus on cellulose-based enzymatic hydrolysis techniques and the use of pulp and paper industry waste. On the other hand, Maqsood et al. [159] conducted research on enzymatically hydrolyzed waste jute fibers to make microcrystals, and then 1, 5, and 10% of the crystals were mixed to PLA polymer to create composite film using a solvent casting technique. When 1 wt.% microcrystal was used, the greatest increases in tensile strength and crystallinity rate were observed over the neat PLA film. Tensile strength can be improved by increasing the amount of interfacial bonding between PLA and microcrystals, increasing the crystallinity of composite PLA, efficient stress transfer at the given reinforcement load, and forming a rigid and percolated network of jute microcrystals (JMC) due to hydrogen bonding between microcrystals.

In the case of CNF production, the delignified and bleached pulps are subjected to mechanical treatments, which include high-pressure homogenizers or microfluidizers, ball millings, steam explosion reactors, high-speed blenders, extruders, and ultrasonic equipment. The main disadvantage is excessive energy consumption. Therefore, it has been demonstrated in a number of studies that when mechanical processes are combined with an enzymatic treatment approach, energy requirements are reduced [160,161]. Few studies have reported experiments in which the nanofibers are entirely produced by enzymatic approach [83,162,163,164].

## 5. Nanocellulose-Based Nanocomposites Processing

Nanocellulose has sparked a lot of attention in the last several decades as a potential reinforcement for various polymeric materials in numerous advanced applications. Nanocellulose are available in a multitude of forms, sizes, surface chemistries, and characteristics, which can be isolated from a variety of sources, including woody/non-woody, tunicates, algae, and bacteria [165,166,167,168,169]. The resulting nanocellulose exhibits varying degrees of crystallinity, aspect ratios, and morphologies due to the differences in biosynthesis of these sources. Yet, nanocellulose has a high aspect ratio, a Young’s modulus of about 114 GPa, and tensile strength of about 6000 MPa, which are equivalent to typically used inorganic fillers that are not biodegradable or renewable [170]. Unlike typical cellulose-based composites, nanocellulose-based composites have a low content of well-dispersed nanocellulose. The fundamental reason is that in order to attain good mechanical qualities, it is not essential to fill the polymer with a large amount of filler [171]. To put it another way, nanocellulose is widely regarded as a promising candidate for the production of high-performance multifunctional composites.

Yet, there are multiple hurdles in this area, and most of them are linked to the technique of nanocellulose incorporation and how to achieve good dispersal of these nanocelluloses in the matrix. The chemical compatibility of the matrix and the reinforcement, as well as the dispersion of nanocellulose in the matrix, are both critical characteristics for the manufacture of excellent nanocomposites [172]. Dispersion is a fundamental component in nanocellulose-based nanocomposites and is of major concern to researchers. The method employed in the fabrication of nanocomposite is determined by the matrix and reinforcement characteristics, but the approach must enable thorough dispersion of the reinforcement in the matrix, which means that processing methods are unique to each case. In a nutshell, the nanocomposite fabrication method and nanocellulose properties are critical components in obtaining a better end product.

### 5.1. Solution Casting

**Figure 7:**
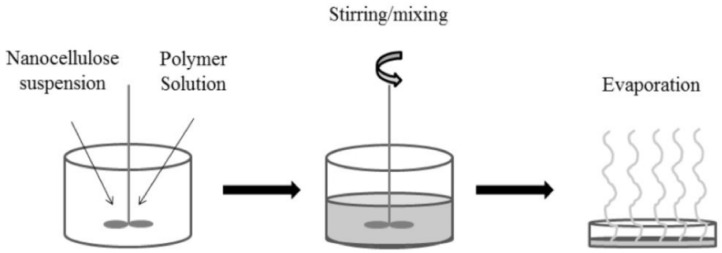
A schematic illustrating the solvent casting procedure to synthesize nanocellulose-based nanocomposites.

One of the most extensively utilized techniques for synthesizing nanocellulose-based nanocomposites is solution casting. The preparation is based on the dispersion of cellulose nanomaterials in solvents, and typically this dispersion is accomplished using sonication, which is essential for achieving effective nanofiller dispersion. The solvent-dissolved nanocellulose suspension is then added to the polymer that has already been solubilized in the same solvent, and the mixture is dried until the solvent has evaporated completely as illustrated in Figure 7. This approach has the advantage of ensuring nanocellulose dispersion, as opposed to other methods.

**Figure 8:**
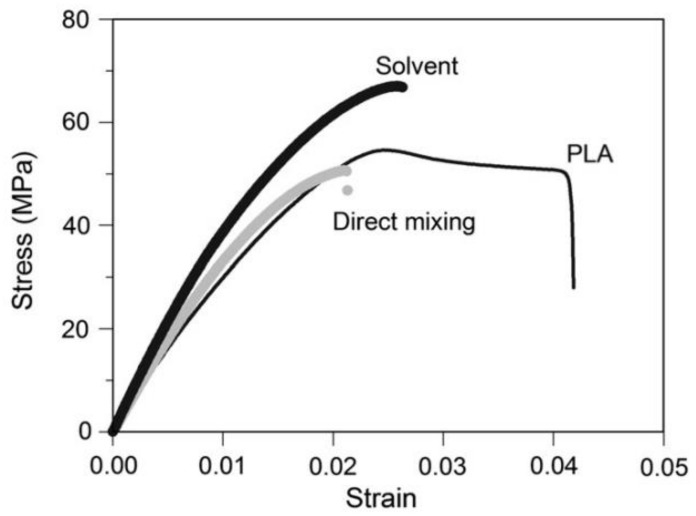
Stress–strain curves for PLA composites prepared using solvent casting and direct mixing methods [176].

The solution casting approach has been used to incorporate nanocellulose into both hydrophobic and hydrophilic polymers including poly(ethylene oxide) (PEO) [173], polylactic acid (PLA) [174] and polyhydroxyalkanoates (PHA) [175] with remarkable results. Iwatake et al. [176] compared the PLA/CNF nanocomposites that have been fabricated using two different methods: casting and melting methods. The findings showed that the samples prepared via solvent casting demonstrated tensile strength than direct mixing and neat samples (Figure 8).

**Figure 9:**
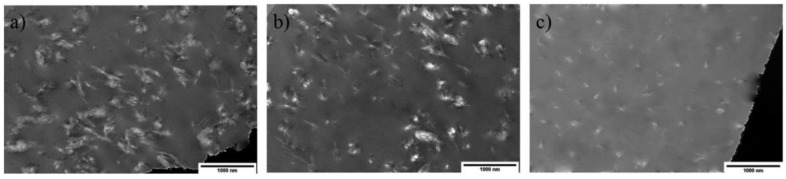
Transmission electron microscopy (TEM) images of PVA/CNC nanocomposites containing 8.3 vol.% CNCs prepared by (**a**) solution casting, (**b**) solution casting and reprocessing in a roller blade mixer, or (**c**) solution casting and reprocessing in a twin-screw extruder. Scale bar = 1000 nm [177].

In another study by Sapkota et al. [177], they compared the mechanical and morphology characteristics of PVA/CNC nanocomposites prepared using solution casting or solution casting followed by torque rheometer processing. From the obtained data, solution casting or solution casting followed by torque rheometer processing produced nanocomposites with better mechanical properties. They also mentioned the most promising way for increasing nanocellulose dispersion is to combine the casting and melting methods. Master batches are often generated by casting CNC into a matrix, for example, through the development of a percolated nanocellulose network. The extrusion process pelletizes the concentrates and dilutes them to lesser concentrations. Figure 9 depicted the TEM images of PVA/CNC prepared using solvent casting and combination methods of solvent casting followed by melt blending and the extrusion process. Figure 9a indicates that the CNCs in solution cast nanocomposites that are well-distributed and appear to form a percolated CNC network, as expected. This morphology agrees perfectly with the mechanical data collected. A similar image can be seen in Figure 9b which the samples were reprocessed using mixer. The CNCs appear to be properly spaced and form an interconnected network in this scenario as well. Surprisingly, the TEM image of samples that had been reprocessed by the extrusion approach showed a drastically altered morphology (Figure 9c). The CNCs in this sample appear to be shorter than those in the parent material (Figure 9a). The creation of an interconnected CNC network cannot be seen; instead, the CNCs appear to be spread out.

### 5.2. Melt Intercalation

Vaia and coworkers [178] reported the melt intercalation procedure for the first time in 1993. This is a top-down approach that entails combining nanoparticles with molten thermoplastic polymers to improve polymer–nanomaterial interactions. Polymer chains infiltrate the reinforcement, which enables intercalation [179]. The combination is then annealed above the polymer glass transition temperature, resulting in a nanocomposite. Since then, it has gained a reputation as the most advantageous and lucrative technique for the synthesis of nanocomposites, owing to its significant potential for use in the polymer industry, where it may be manufactured with compounding devices such as extruders or mixers [180].

The melt intercalation method is more versatile, does not require solvents or chemical reactions, and improves matrix filler interactions by lowering interfacial tension The matrix reinforcement interactions are improved by using an environmentally friendly and simple melt intercalation technique [181]. Intercalated nanocomposites or exfoliated nanocomposites can be developed depending on their compatibility (filler and matrix). In-situ polymerization or adsorption procedures can be employed to generate nanocomposites if the polymers are not even suitable for solution intercalation. In a nutshell, this approach is more adaptable and does not necessitate the use of chemical reactions or solvents.

### 5.3. Impregnation

The basic idea behind the preparation of structural composite materials is to impregnate a fiber reinforcement with a liquid resin and then consolidate or cure the fiber-resin system under controlled temperature and pressure [182]. Throughout the impregnation process, the resin is introduced to the preceding dry fiber, ensuring that the fiber is adequately saturated. This process results in a lamina. Various methods are used to achieve thorough and consistent impregnation, although automation is the most common today, with a compaction roller ensuring that the resin flows evenly throughout the fibers. During the filament winding process, for example, fibers are impregnated with a resin solution. In the hand lay-up technique, materials that have already been impregnated by the material provider in a controlled setting are used. In the automated fiber insertion process, this phase is carried out at the impregnation lines. The impregnation line produces prepreg. Prepregs are composite materials that have a reinforcement fiber pre-impregnated in a certain ratio with a thermoplastic or thermoset resin matrix. As they are cured at high temperatures and pressures, prepregs have special features [183].

Baché et al. [184] mentioned the initial step of the manufacturing process is a complicated issue with numerous conceivable final saturation profiles due to the various processes and materials that can be applied. Process parameters (e.g., pulling velocity) and materials characteristics (e.g., slurry viscosity and fabric geometry), for example, have a significant impact on impregnation, with each set of slurry and fabric qualities necessitating a certain pressure and impregnation duration. The impregnation process has a significant impact on the mechanical qualities of the final product. Managing macro-porosity within the material and guarantee adequate cohesion between fibers and matrix ensures that the fiber tows are fully impregnated with the slurry during impregnation.

Nonetheless, one of the key problems of thermoplastic matrix composites is that thermoplastic melts have a higher viscosity than thermoset melts, making impregnation more challenging [185,186]. Reducing the flow length through through-thickness impregnation [187], using low viscosity engineering thermoplastics [188], or thermo-consolidating fiber-matrix premixed semi-products are some of the potential solutions for reducing the manufacturing cycle time of structural thermoplastic composites while maintaining high quality of the final part. The stacking of polymer film between fiber plies, the powder impregnation of bundles, or the commingling of polymer and reinforcing fibers inside the yarns are some of the existing technologies that use pre-mixed semi-products.

### 5.4. In-Situ Polymerization

In-situ polymerization is another effective method for dispersing cellulose nanoparticles uniformly in the matrix, resulting in a strong interaction between the matrix and the filler. Nanocelluloses are mixed in a neat monomer (or many monomers) or a monomer solution, then polymerized in the presence of the dispersed nanocellulose [189]. As the monomer is usually very fluid, impregnation can be conducted without disrupting the nanocellulose arrangement excessively. The monomer plays a dual role in this method, acting as both a dispersion for the nanocellulose and a matrix precursor for in-situ polymerization. Following polymerization, the material is processed with only minimal finishing needed [190]. A curing agent or peroxide is added to thermosets such as epoxies or unsaturated polyesters to induce polymerization, whereas thermoplastics can be polymerized either by adding a curing agent or increasing the temperature. Many findings on in-situ polymerization methods have demonstrated that covalent linkages between the matrix and nanocellulose form in the final nanocomposites.

In-situ polymerization enables for direct integration of well-dispersed nanoparticles in bulk polymer composites, whereas film casting procedures are only suited for the fabrication of thin films, and melt processing methods frequently involve poor particle dispersion [191]. In addition, in-situ polymerization can be utilized to make nanocomposites, as polymers are not suitable for melt processing due to their low temperature stability, nor for solution casting due to their insolubility in any solvent. Motaung et al. [192] synthesized nylon/CNC nanocomposite by three different methods: melt compound, in-situ polymerization, and solvent technique. The in-situ technique was found to be the most effective since the CNC is introduced directly to the monomer and has time to scatter well before the initiator is added to begin polymerization. The in-situ approach allows for precise particle size and morphological control.

Furthermore, nanocellulose is highly compatible with the PU matrix since it is highly hydrophilic, hence the in-situ polymerization process can improve interaction and bonding even more. Cao et al. [193] successfully synthesized a series of waterborne polyurethane (WPU)/CNC composites using in-situ polymerization. The conditions were optimized to promote the grafting of a part of the pre-synthesized WPU chains onto the surface of CNC and the resulting nanocomposites were cast and evaporated. Some of the pre-synthesized WPU chains were successfully grafted onto CNC through the interaction between the iso-cyanates of the WPU prepolymers and the hydroxyls of CNC. These grafted-WPU chains were able to establish a crystalline structure on the surface of CNC, causing the matrix to crystallize, and resulting in a co-continuous phase. As a result, efficient dispersion and strong interfacial adhesion between CNC and WPU were achieved, considerably improving the thermal stability and mechanical properties of nanocomposites. WPU/CNC nanocomposites with improved characteristics could be used in a variety of consumer goods, including elastomers, foams, paints, and adhesives.

Nevertheless, the main downside of this approach is that as the polymerization process continues, viscosity increases, making handling difficult and restricting load fraction [194]. This method can only be used if the polymerization takes place in a liquid phase and nanocellulose can be disseminated in the polymerization media. Another critical problem is the compatibility of nanocellulose with the matrices. Due to its high hydrophilicity, nanocellulose is difficult to dissolve in hydrophobic monomers such as acrylics, resulting in weak interfacial interaction with hydrophobic matrices [195]. Additionally, since it needs a low elastomer viscosity, in-situ polymerization is still not a preferred process for the production of specific sets of materials [196].

### 5.5. Coating

Coatings form thin layers that are either external or packed between two substrates, resulting in a composite structure. The thickness of the layers ranges from a tenth of a nanometer to a micrometer [180]. The coating process, which involves spreading a slurry onto the surface of a variety of reinforcing/supporting materials, is widely employed in food packaging, particularly for paper and paperboard packaging materials, which are typically coated to increase moisture, gas, and grease-barrier characteristics. The particles form a film layer that adheres to the material surface during the coating process, whilst excess liquid medium is evaporated [197]. Thin coating layers of nanocellulose alone or in combination with other polymers can be developed due to the high dispersion of nanocellulose in water. A range of coating methods, including aqueous, solvent-based, extrusion, and electrospinning coating, can be used to apply nanocellulose-based layers on cellulosic-based materials or synthetic films [198].

Ansari et al. [199] developed nanocomposite coatings consisting of CNF, (3-glycidyloxypropyl) trimethoxysilane (GPTMS) and tetrapropyl zirconate (TPOZ) in a low temperature, scalable process based on spraying of a colloidal dispersion followed by drying and curing. The coatings are highly multifunctional, combining high hardness and fracture toughness with outstanding optical transmission. Moreover, the hydrophobic surface functionalities of these thin coatings result in a high-water wetting angle (>90°). With 20 wt.% nanocellulose, the most significant increases in coating characteristics were achieved. Koppolu et al. [200] combined PLA and nanocellulose into thin multilayer coatings onto a paperboard using a pilot-scale extrusion coater. They found that the water vapor transmission rate for nanocellulose + PLA coatings was lower than that of the control PLA coating even at a high relative humidity of 90% (38 °C). The multilayer coating showed a 98% lower oxygen transmission rate and a 99% lower heptane vapor transmission rate as compared to the PLA-coated baseboard. The grease barrier for nanocellulose + PLA coatings improved five-fold and two-fold, respectively, when compared to nanocellulose alone. This approach of processing nanocellulose and PLA into several layers using a slot-die and extrusion coating system has the potential to develop a biobased and biodegradable barrier packaging paper. In another study, Arrieta et al. [201] used the electrospinning techniques to fabricate bionanocomposites based on PLA-PHB/CNC. The addition of cellulose nanocrystal (CNC) to mats improved their mechanical and thermal resistance as well as their water resistance.

The benefits and drawbacks of each processing technique used to fabricate nanocellulose-based polymer composites were summarized in Table 6. Nanocellulose-based materials must be processed thoroughly since this influences their applicability. The fundamental difficulty, as with any nanoparticle, is in ensuring that they are evenly distributed within a polymeric matrix. The polymer melt approach is most likely the easiest processing method out of all of them because it is an environmentally friendly procedure that is both commercially and economically feasible. However, the intrinsic incompatibility of cellulose with the majority of polymeric matrices and problems with thermal stability make it difficult to identify the optimal conditions.

## 6. Nanocellulose Based Nanocomposites for Sustainable Applications

Nanocellulose is used in a variety of fields, including medicine, packaging, cosmetics, electronics, food, automotive, optical materials, aerospace, and other fields [26,134,136,202,203,204,205,206]. Among its distinctive features are its hygroscopicity and chemical inactivity. Nanocellulose could also be used in a variety of industries due to its lack of high sorption and toxicity [40,41]. In addition to its inexpensive production costs, nanocellulose possesses remarkable characteristics that make it attractive for extensive adoption, including excellent mechanical properties, fatigue resistance, adequate strength, and light weight [129,130,136,207,208,209,210]. Additionally, cellulose has an excellent water-holding capacity, making it ideal for usage in biocompatible coatings and medication discharge, formulations, scaffolds and wound dressing. In terms of the thermal conductivity characteristics of CNF, Adachi et al. [211] reported the experimental study of individual CNFs using the well-established thermal bridge method. The results obtained show thermal conductivity of individual CNFs is found to be approximately 2.2 (±1.2) W/m K at 300 K, and the temperature-dependent data from 40 to 320 K indicate that the phonon transport of CNFs is dominated by boundary scattering. Theoretical simulation results on thermal conductivity of individual CNFs and cellulose bulk crystal support the experimental results and suggest that intermolecular interaction also impedes the thermal transport.

Another application of nanocellulose to highlight is a study on utilization of CNF cellulose nanofiber films for smart heat dissipation by convection [212]. In this study, the *kirigami* (the traditional art of paper cutting) with a thermally conductive CNF film to propose a flexible cooling system through convective heat dissipation has been used. By stretching the *Amikazari* (net decoration) pattern produced by *kirigami* and allowing air convection through its aperture at 3.0 m/s, the thermal resistance was reduced to approximately one-fifth of that without *kirigami* and convection. The *kirigami* spaces defined the outlet air velocity, resulting in a significant increase in the heat-transfer coefficient. The *kirigami* heat dissipation concept enables the design of electronics using a variety of film materials as shape-variant cooling structures, which will move a wide range of thermal engineering and electronics applications. Reshmy et al. [213] stated that the anticipated markets for nanocellulose can be categorized into three levels: (1) high-end applications (like batteries, printed electronics, and paper-based value-added materials); (2) mid-range applications (including structural materials, food); and (3) low-end, widespread pulp and paper commodity applications (like lighter and stronger conventional paper products). Table 7 listed the key applications and potential benefits of nanocellulose [213].

### 6.1. Food Packaging

The majority of modern food packaging materials are synthetic and non-biodegradable, prompting environmental concerns about the accumulation of plastics in landfills and waterways. The food sector is increasingly in need of environmentally friendly and sustainable materials that meet food-packaging specifications. However, most of the polymer materials pose poor water resistance and mechanical strength that should be improved for food packaging application. The protection provided by the package is a crucial component of the preservation process for the majority of food products. The specifications of a packaging system for fresh, frozen, dehydrated, thermally processed, or aseptic products are determined by: (i) the intrinsic qualities of the food product, such as water activity and oxidation potential, which determine their perishability; (ii) extrinsic factors, such as storage temperature, relative humidity, and exposure to light; and (iii) the required shelf-life. When defining the necessary barrier ability to water vapor, oxygen, and other gases, including odors and light, all these elements must be taken into consideration [214].

Alternatives are being sought in light of the expanding economic and environmental problems around these materials. The advantages of packing materials made of cellulose, in particular nanocellulose, have recently been thought of [32,136,205]. Evidently, long-term usage of such by-products could assist in resolving the difficulties in industry with costs, sustainability, and disposal. Nanocellulose is a type of renewable resource that has the potential to address sustainability problems. In the expanding commercialization of nanotechnologies, nanocellulose is extremely important. As a result, researchers and industrialists are examining and investigating novel nanocellulose manufacturing techniques and applications. From the perspective of packaging, there are several reasons to use nanocellulose, including edibility, flexibility, biodegradability, transparency, antimicrobial, barrier and mechanical characteristics.

**Figure 10:**
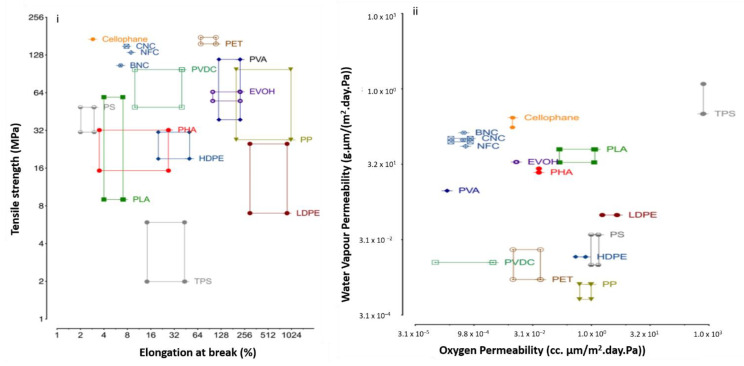
(**i**) Mechanical and (**ii**) barrier properties of conventional plastics packaging versus nanocellulose. Reproduced from ref. [215].

Biopolymers such as polylactic acid (PLA), polyhydroxyalkanoates (PHA), and thermoplastic starch (TPS) have been explored as alternative options for non-biodegradable plastic materials subject to worldwide concern. Silva et al. [215] compares the mechanical properties, i.e., tensile strength, elongation at break and barrier qualities, i.e., water vapor and oxygen permeability of conventional plastics and some biopolymers including PET, PVA, TPS, LDPE, HDPE, PLA, EVOH, as shown in Figure 10. From the analysis, they found out that biopolymers have lower elongation at break and greater water vapor permeability than standard plastics. However, the existing bio-polymer materials lack the performance to meet the most stringent specified criteria for food packaging such as inherent food product properties, extrinsic factors, and shelf life. To increase the performance of the biopolymers for food packaging applications, nanocomposite technology has been considered as a possibility. Plant nanocellulose is considered as a renewable and sustainable source with competitive features that can be used in food packaging manufacture based on its mechanical and barrier properties. Antimicrobial and antioxidant characteristics are two additional functions of employing plant nanocellulose as a material for packaging. Furthermore, nanocellulose is a preferred material for use as a support in active and intelligent packaging. Nanocellulose has grown in popularity as a food packaging component due to its high rigidity, low oxygen content and permeability, as shown in Figure 10 as CNC, BNC and NFC. As nanocellulose may act as a carrier for some active ingredients, such as antioxidants and antimicrobials, it is also able to increase food quality while also extending the shelf life of foods.

Srivastava and co-workers [216] used a solution casting approach to synthesize PVA–banana pseudostem fiber (BPF) nanocomposite films that were reinforced with increasing amounts of nanocellulose filler to improve mechanical and barrier properties. The mechanical characteristics of films reinforced with 3 wt.% nanocellulose improved by 14.3% in tensile strength, but the elongation at break decreased by 9.1%. The amount of nanocellulose added decreased the water vapor permeability by 29.7%. The water swelling test revealed positive results, with the film regaining just 16.8% of its weight after 24 h of immersion in water, compared to 67.4% for PVA composite films containing no nanocellulose, making them a viable alternative to plastic packaging films.

Asad et al. [217] developed polyvinyl alcohol (PVA)-based nanocomposite films incorporating TEMPO-oxidize nanocellulose (TONC) suspension with a content ranging from 0.5 to 6% (*w*/*w*). It was found that the 4% (*w*/*w*) TONC content reinforced nanocomposite had the maximum tensile strength and modulus, with increments of 122 and 29%, respectively, while the elongation at break was reduced by 42.7%. The addition of TONC increased the thermal stability of PVA-based nanocomposite films and boosts its crystallinity due to strong linkage between the hydroxyl groups of the materials, while significant losses are found beyond 2 wt.% loading. The melting temperature peaks and enthalpy of nanocomposite films are significantly reduced when TONC is added beyond 2%. In another study conducted by Zhang et al. that reinforced CNF extracted from *Enteromorpha prolifera* into PVA matrix, they found that the light transmittance of PVA/CNF composite film could reach more than 94%, and the swelling degree was 250%, which is 29% lower than that of the pure PVA film, and the water resistance was improved when the CNF content was 0.1 wt.%. Furthermore, the tensile strength, elastic modulus and toughness were 99.1%, 1547% and 117.9% respectively, which are higher than those of neat PVA.

Patel and Joshi [218] fabricated composite films by the incorporation of CNF from banana waste into a PVOH matrix. They reported that at 2% CNF in PVOH, the solubility of composite film was 92.54%; beyond this composition, the solubility film falls, which may limit its application as a soluble packaging material. At 2% CNF addition in 10% PVOH film, the tensile strength was examined to be 2.36 kgf with a Young’s modulus of 59.16 N mm^−2^. Further addition of CNF to PVOH resulted in a significant reduction in the tensile strength. Hence, it has been demonstrated that PVOH/CNF film can be used as a soluble packaging material. Additionally, researchers have developed a strong interest in active and intelligent packaging, and many studies in this area are published annually. An active packaging system, such as one with antibacterial and antioxidant capabilities, absorbs or releases compounds to prolong the food’s shelf life. Intelligent packaging provides information, such as on the freshness of the food, by monitoring the state of packaged food or the surroundings. In terms of these activities, nanocellulose is inert, but it makes a great support for compounds that might have an active or intelligent function in the food packaging system [214].

By impregnating BNC composite films with spherical flavonoid silymarin (SMN)-zein nanoparticles, effective antioxidant activities were demonstrated. These qualities were maintained for at least 72 h owing to the delayed release of the active ingredient. For *Staphylococcus aureus*, *Escherichia coli*, and *Pseudomonas aeruginosa*, the antibacterial activity of the films demonstrated an inhibition ratio of 60, 20, and 30%, respectively. The system was then tested using salmon fish, which demonstrated quality indicators of thiobarbituric acid reactive substance assay values that were 40% and 30% lower than those of the control, respectively [219]. Ferulic acid and feruloylated arabinoxylo-oligosaccharides were used to functionalize arabinoxylans-based nanocomposite films that contained 50% CNF during solvent casting. The films demonstrated antioxidant activity of up to 90% in the 2,2-diphenyl-1-picrylhydrazyl hydrate assay. In addition, the film demonstrated bactericidal activities with a 3-log CFU mL^−1^ decline against *Staphylococcus aureus*, bacteriostatic activity against *Escherichia coli*, and antifungal efficacy towards the polymorphic fungus *Candida albicans* with a 1.1-log CFU mL^−1^ reduction [220].

It has also been documented that nanocellulose has been functionalized for intelligent food packaging, particularly for the development of freshness indicators with the intention of detecting food spoilage. Freshness indicators typically assess changes in pH or gas composition inside the packaging, and these changes are translated into a color response, which is simple to quantify and may be linked to the food’s freshness [221]. Indicators for freshness were developed by Kuswandi et al. [222] and Lu et al. [223] using BNC-methyl red and TEMPO-mediated NFC hydrogels with a bromothymol blue/methyl red mixture, respectively. The composites responded to the amount of volatile biogenic amines, and it was discovered that the CO_2_ levels increased as the chicken spoiled, changing color as a result of the pH adjustment.

**Figure 11:**
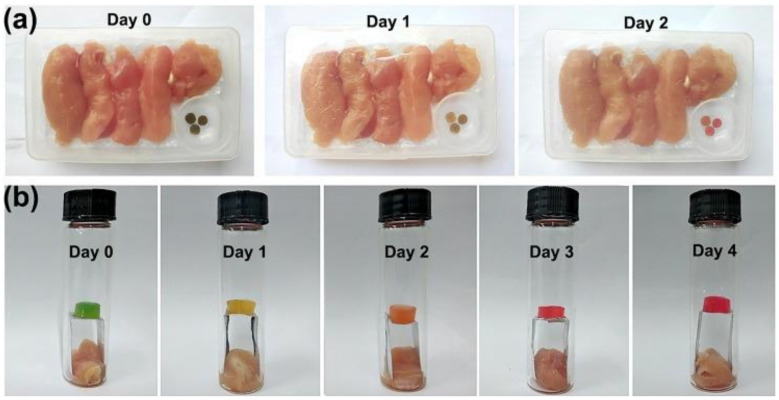
(**a**) Indicator hydrogel for applications in intelligent food packaging; (**b**) Relationship between color of indicator hydrogel and freshness of chicken breast. Reproduced from ref. [223].

The fabricated bagasse nanocellulose-based hydrogels for intelligent packaging with a freshness indicator for monitoring spoilage of chicken breast [223]. In this work, by using TEMPO-mediated oxidation to create nanocellulose from sugarcane bagasse cellulose filaments, a stable self-standing nano-hydrogel matrix was created with an aid of Zn[2]^+^ cross-linking. The pH-responsive dyes were carried by the nano-hydrogel that act as a freshness indicator for chicken breast in packaging. As the freshness of the chicken meat was indicated by observing variations in CO_2_ levels inside the chicken meat packing, the hydrogel indicator was found to be responsively sensitive to CO_2_, where the color changes in response to the increases of bacteria growth [224] were as shown in Figure 11.

### 6.2. Biomedical

Nanocellulose has attracted a lot of attention for its application as a biomedical material, specifically in tissue engineering, drug delivery, cartilage replacements, tissue engineering, cardiovascular applications, wound dressings and medical implants in recent years due to its exceptional physical qualities, particularly surface chemistry, and outstanding biological properties, such as low toxicity, biocompatibility, and biodegradability [225]. It opposes the nature of proteins and prevents hostile tissue reactions; its structure lessens hemolytic and immunogenic reactions. Additionally, it promotes both tissue contact and development. Yet, nanocellulose degrades relatively slowly in both in vivo and in vitro conditions, making it appropriate for long-term applications. It can support heavy loads and has a high wear resistance, making it suitable for use as a scaffold in long-term applications [226].

Siqueira et al. [227] synthesized different nanocellulose-alginate hydrogels containing cellulose nanocrystals, TEMPO-oxidized cellulose nanocrystal (CNCT), cellulose nanofibers or TEMPO-oxidized cellulose nanofiber (CNFT). The morphological and mechanical properties as well as non-cytotoxic behaviour of the CNFT-alginate gels enhanced the bioadhesion, growth, and proliferation of the cells onto the gels. Alginate nanocellulose gels could therefore be used in the field of tissue engineering for purposes including tissue repair or wound healing. In order to ensure perpendicular growth of bacterial nanocellulose produced by *Komagataeibacter xylinus* E25 in stationary culture, Ludwicka et al. [228] developed composites that were produced in specially designed bioreactors outfitted with a set of perforated mesh stripes threaded vertically to the culture medium. The in vitro inflammatory reactions of the produced biocomposites were examined, and they included mast cell degranulation with N-acetyl-d-hexosaminidase release and mast cell adhesion. The obtained results show that, following the culture and purification processes, the components of the composites are well integrated. When bacterial cellulose is added, the composites become less immunogenic than polypropylene alone.

Even though nanocellulose has the potential to accomplish better-than-conventional polymeric materials in biomedical applications, designing nanocellulose-based biomaterials for wound healing still demands controllability over biophysical and biochemical signals to assist a variety of cellular activities in healing processes. Liu et al. [229] developed bioactive CNF scaffolds with regulated release of basic fibroblast growth factors (bFGFs) for conceivable uses in the treatment of wounds. According to the Quartz Crystal Microbalance with Dissipation Monitoring (QCM-D) analysis, the polyion complex interaction between the positively charged bFGFs and the negatively charged CNF resembles the interaction between bFGFs and heparin sulfate in the extracellular matrix in the body. This connection allows for the possible protection of bFGFs against denaturation in addition to storing it in a form that is easily accessible and from which it is progressively released. By adjusting the CNF surface chemistry and deconstructing the scaffolds in situ, the release profile of bFGFs from the CNF scaffolds was customized. The in-situ enzymatic deconstruction of the scaffolds offers a chance to regulate the bioavailability of bFGFs for cell growth and proliferation, but more critically, to balance the destruction of the scaffolds and the synthesis of new tissue in wound healing. As demonstrated by the MTT assay for cell proliferation and fluorescence imaging of cells cultured in 3D scaffolds, CNF scaffolds loaded with bFGF can significantly promote cell proliferation, even when only a minimal amount of bFGF is loaded. The bioavailability of bFGF is further increased by enzymatic deconstruction of the CNF network, which further encourages cell division. Their research could be a significant step towards the development of nanocellulose-based biomaterials with specific biophysical and biochemical cues for wound healing and other biological applications.

### 6.3. Automotive and Aerospace

In addition to exceptional mechanical strength, multi-functional nanocellulose-based nanocomposites also exhibit remarkable combinations of thermal, electrical, optical, and magnetic properties. The mechanical and physical properties of nanocomposites are thought to be significantly influenced by molecular-level interactions between nanocellulose and polymer matrix as well as the presence of a very extensive nanocellulose–polymer interfacial region. It has developed into a group of polymeric materials with improved mechanical properties, increased modulus and dimensional stability, flammable retardation, better scratch and marine resistance, excellent thermal and process characteristics, reduced part warping, and increased impact resistance, making it nearly possible for metal replacement in automotive applications.

The first nylon-6/clay biocomposites were introduced to the market in 1991 by Toyota Motor Co. (Toyota City, Japan) to produce timing belt covers for the Toyota Camry engine in collaboration with the Ube industry. This marked the beginning of the commercial exploitation of polymeric composites. Approximately at the same time as the Mitsubishi GDI 3 (Tokyo, Japan) engines were being produced through injection molding, Unitika Co. of Japan introduced nylon-6 nanocomposite for engine covers. This product appears to offer a 20% weight reduction and a durable surface finish. In 2002, working with Basell (now Lyondell Basell Industries, Rotterdam, The Netherlands), General Motors (Detroit, MI, USA) introduced a polyolefin component for the GM Safari and Chevrolet Astro vans (Detroit, MI, USA) that was gradually covered with 3-percent nanoclay. These nanocomposites were then used for the doors of the Chevrolet Impala [213]. Using nano-enhanced sheet molding materials created by Molded Fibre Glass Companies, Ohio, General Motor (GM) manufactured a single piece rear-floor molded compression assembly for its Pontiac Solace in 2009. This is also applied to GM’s Corvette ZO and Corvette Coupe models. Polymer nanocomposites may be advantageous for the automotive industry in a variety of applications, including engines and power trains, suspension and braking systems, exhaust systems and catalytic converters, frames and body parts, tires, coatings, lubricants, and paints, as well as electrical and electronic equipment. Since then, extensive research on nanocomposites has been conducted on a global scale.

Sanoj and Balasubramaniam [230] developed high performance structural nanocellulose composites for motor vehicle spring suspension systems. In the hybrid composite system, nanofibers are evenly distributed across the system region. Through a shear deformation process, the 3D hierarchical configurations of jute fibril reinforcements in the epoxy matrix system efficiently contribute to the polymer matrix toughening and crack propagation resistance. The hybrid composite system delamination resistance and fracture toughness are greatly improved by these nanofibrils, which serve as a connecting bridge between the glass fiber laminate. This important feature enhances the hybrid composite leaf spring strength, rigidity, and dynamic response when compared to a glass fiber-reinforced composite suspension system.

Other than the automotive industry, Duzik et al. [231] reported that currently CNFs are being developed for use as reinforcement materials in aerospace structural composites to increase adhesion, thermal stability, and mechanical stability. The application of CNF can significantly lower aircraft weight, resulting in reduced fuel consumption. Another CNF structural application is in the NASA HELIOS heliogryo solar sail blade material, which demands for a thin coating of less than 3 µm. Moreover, the small fiber diameter of nanocellulose, like other advanced fiber architectures, allows for the implantation of sensors such as Fibre Bragg grating (FBG). With this technique, anyone may identify object damage by retrieving data at a near distance from the sensor. As a result, the aerospace industry may be able to use nanocellulose aerogels as a novel option to composite structures [232].

### 6.4. Sensor

An ideal sensor must be simple to make, affordable, and able to detect analytes quickly with good sensitivity and selectivity. Nanocelluloses are widely accessible, biocompatible, and incredibly adaptable. Due to their enormous surface area and many functional groups, nanocellulose can also interact with a variety of molecules. These elements work together to make nanocellulose appealing for use in sensing applications. Nanocellulose is typically employed as one of the components, together with other stimuli-responsive chemicals, for making sensors [233]. Numerous nanocellulose-based sensing devices showed receptiveness to a variety of stimuli. The most common way that nanocellulose is modified to acquire different properties and functionalities throughout its surface is by combining it with other superficial groups, metal oxide, polymer chains, or even other nanoparticles. This results in low-cost smart materials that are sensitive to biotechnology. This recently developed nanocellulosic material was an excellent choice for numerous sensing system applications.

A green, flexible photonic device for sensing purposes was created by Jaiswal and colleagues using nanocellulose [234]. To develop thermoresponsive hybrid films, CNF and cellulose nanocrystals were combined with a black thermochromic pigment as matrix materials. An all-optical modulation device was demonstrated by making use of the produced film’s thermoresponsive nature. A 660 nm visible diode laser was used to modulate continuous infrared light (1300 nm). They found that films underwent a localized thermochromic change as a result of the laser intensity. A homogeneous cyclic modulation depth of 0.3 dB was attained when the laser was pulsed at 0.3 Hz. From this research, functional nanocellulose hybrid films can be used as a light switch (modulator) in a number of thermally stimulated sensing applications, including temperature monitoring, energy conservation, and counterfeit prevention.

Other than in film forms, when sensors are in the form of highly porous aerogels, the benefits of the high nanocellulose surface area in sensors can be increased. Among all, CNFs are an excellent candidate for producing sensors because of their high porosity and excellent aerogel-forming ability [233]. A lightweight conductive supramolecular aerogel with hierarchically porous 3D structures (porosity of 96.9%) was invented by Wang et al. [235]. This aerogel has excellent capacitance retention, a high conductivity of 0.372 S/cm, and a larger area-normalized capacitance (C_s_) of 59.26 mF/cm^2^ than other 3D chemically cross-linked nanocellulose aerogels by 20 times. By incorporating the electrode material in lightweight supercapacitors and wearable flexible devices, the supramolecular conductive aerogel might also be employed as an all-purpose sensitive sensor for poisonous gas, field sobriety tests, and health monitoring devices. Recent developments in nanocellulose-based composites for sensing applications are listed in Table 8.

### 6.5. Water Purification Treatment

Aquatic ecosystems decline as a result of human-caused water contamination. This limits access to pure water, which is the main cause of death worldwide. Due to its inexpensive cost, high aspect ratio, innate environmental inertness, and huge natural abundance, nanocellulose can be used for water purification. Nanocellulose materials offer great potential for becoming an efficient absorber of contaminants from wastewater. Additionally, the nanocellulose effective functional surface permits the addition of chemical amounts that could improve the ability of pollutants to bind to the nanocellulose. The most studied way to improve the sorptive effectiveness of nanocellulose is probably through carboxylation. According to Yu et al. [250], adding succinic acid moieties to CNC significantly increased the binding effectiveness of aqueous solutions to Cd^2+^ and Pb^2+^. Their capacity to remove these harmful heavy metal ions was further enhanced by the conversion of carboxylic acid groups into sodium carboxylates.

Goswami et al. [251] combined chitosan and nanocellulose from sugarcane bagasse to develop the nanocomposite membrane. The nanocomposite membrane was evaluated for its ability to remove chromium ions from aqueous solution (100 ppm) using a vacuum filtering device. During the investigation, a total of 10 cycles were run to assess the chitosan/nanocellulose composite membrane’s ability to remove chromium ions. The first four cycles dramatically reduced the concentration of chromium ions, which ranged from 87 to 29 PPM. The swelling study (5 min) and chemical stability (24 h) were also evaluated where the obtained results revealed that a maximum swelling ratio of 1.8 was achieved within 5 min, whereas the membrane is completely dissolved in acidic medium and swells in basic medium.

In another study, cellulose nanowhiskers (CNW)-filled hydrogel composites based on chitosan-g-poly(acrylic acid) matrices were constructed, and their capacity to adsorb Pb(II) and Cu(II) ions from water was examined [252]. The optimized parameters showed that utilizing 20 mg of the hydrogel composite containing 10 *w*/*w*% of CNW, the highest adsorption of Pb(II) (818.4 mg/g) and Cu(II) (325.5 mg/g) was obtained in 30 min at pH 4.0. Functional groups included in the hydrogel matrix and CNWs serve as coordination sites for the adsorption, as determined. According to desorption experiments, the post-utilized hydrogel composite can be renewed and employed once again in different adsorption processes without significantly losing effectiveness. In order to obtain adsorbent materials for real-world applications, these insights may be useful.

A bio-based and water-resistant composite aerogel made of two kinds of cross-linked renewable nanofibers was created by Sorriaux et al. [253]. Through the process of periodate oxidation, CNFs coated with poly(dopamine) and amyloid protein nanofibers are cross-linked to form a double network. The resulting aerogel has a high capacity for adsorbing pollutants and good mechanical strength. The composite aerogel was effectively used to demonstrate the removal of dyes (rhodamine blue, acriflavine, crystal violet, malachite green, acid fuchsin, and methyl orange), organic traces (atrazine, bisphenol A, and ibuprofen), and heavy metal ions (Pb(II) and Cu(II) from water. Moreover, the bio-based aerogel particularly showed good adsorption efficiencies for crystal violet (93.1% in 30 min), bisphenol A (91.7% in 5 min), and Pb(II) ions (94.7% in 5 min), respectively. Additionally, the aerogel ability to adsorb and desorb Pb(II) ions shows that it has a large capacity for reuse because it consistently maintains effective removal rates. The findings imply that this type of strong, bio-based composite aerogel is a viable adsorbent for effectively and sustainably purifying water of a variety of pollutants.

### 6.6. Electronic and Optical

Energy-intensive technologies used as technological advancement are gaining attraction. In the case of disposal methods, this could have an impact on the environment. Resource depletion is driven by the use of non-biodegradable plastics, materials produced from petroleum in the production of energy gadgets, and the excessive use of rare elements like gallium and indium [254]. Currently, research is being conducted to introduce nanocellulose applications in electro-active materials such as dielectric materials, electrically conductive materials, microelectronic components, etc. The utility of nanocellulose in electrical applications can be attributed primarily to its flexibility, piezoelectric and dielectric properties, and durability qualities that are similar to those of other bio-derived materials. As nanocellulose is naturally nonconductive, it can be modified with nanocarbon, conductive polymers such as polyaniline (PANI), polythiophene (PTh), polypyrrole (PPy), polyacetylene, and poly(phenylenevinylene), or metallic particles. They can be applied in energy storage devices like Lithium-ion batteries or super capacitors as well as green electronic components like transistors, antennas, and touch screens [255].

CNF film is a viable option for a transparent insulating or semiconducting substrate since it is an inexpensive and environmentally friendly substrate in the semiconductor industry. The invention of transistors made of paper has made low-cost, flexible, and disposable microelectronics a possibility [256]. In conjunction with printed electronics, cellulose modification and the proper production technology will open up a new path for electronics made of cellulose paper, or “papertronics” [257,258]. Han et al. [259] reported a novel type of electroconductive hybrid elastomers based on a natural rubber (NR) matrix and nanostructured CNF-polyaniline (PANI) complexes that combine the conductivity of PANI with the function of CNF as a biotemplate. The CNF-PANI complexes with ideal dispersity and high aspect ratio are developed by in-situ oxidative polymerizing aniline monomers on the surface of CNF templates. These complexes are then uniformly dispersed into NR latex to generate CNF-PANI/NR elastomers with a hierarchical 3D network structure through a latex coagulation process. The nanocomposite showed good stretch ability, low density, optimum conductivity, increased mechanical characteristics, and inherent flexibility. This technique encourages the advance uses of bio-based materials, including CNFs and NR, in potential soft electronics, such as strain sensors and flexible electrodes, by synthesizing multifunctional elastomers in a simple, scalable, and environmentally friendly approach.

Inui et al. [260] produced a high-dielectric-constant (k) of high density cellulose nanopaper composite by mixing a small amount of silver nanowires with CNF in order to produce a high density cellulose nanopaper by increasing the pass number of the fibers (collision number from several 10 μm to about 15–40 nm after 10 passes). A small amount of silver nano wires was dispersed inside the cellulose nanopaper, and keeping them non-percolative dramatically increased the k value of the nanopaper and retained its flexibility. The k value increased with increasing nanopaper density. The cellulose nanopaper contained a densely packed nanostructure, with a density of up to 1.3 g cm^−3^ and k value of up to 5.3. This study also compared the k values of the fabricated nanopaper with other substrates and the result shows that the traditional paper substrates made from untreated cellulose pulp fiber with micrometer-scale widths exhibited lower k values (k = 2.9 at 1.1 GHz) than typical plastic films such as polyethylene terephthalate (PET) (k = 3.1), polyethylene naphthalate (PEN) (k = 3.4), and polyimide (PI) (k = 3.4). The experiment also found that the low k value of traditional paper may be because of its porous microstructure of micrometer-sized cellulose fibers.

He et al. [261] created cellulose nanofiber/polyaniline (CNF/PANI) composite films using in-situ polymerization of aniline in a nanocellulose solution that was extracted from bamboo fiber. They concluded that the CNF/PANI composites’ mechanical and electrical conductivity results suggested that they may find usage as biological sensors, electromagnetic radiation shielding materials, and anti-static materials. Nanocellulose/graphene oxide electrolytes were also developed as flexible rechargeable zinc–air batteries by Zhang et al. [262]. The nanocellulose improved the electrolytes by providing excellent structural stability, high water content, low anisotropic swelling degree, and superior ionic conductivity.

According to Mishra et al. [263], films with well-arranged CNC may display piezoelectric effects resulting from the combined yield of the individual CNC. The piezoelectric effect increased with the increasing degree of alignment observed, and the orientation of the CNC consequently affected the film’s piezoelectricity. Ultra-thin films with various levels of CNC alignment can produce varying rates of electro-mechanical actuation, which may be employed in applications like ultra-sensitive micro balances. The high degree of crystallinity makes it possible for CNC to alter polarization densities. Additionally, due to its dielectric qualities, it can be employed in a variety of applications as a useful insulating material.

The development of electronic skins for uses including biomedical sensors, robotic prosthetics, and human–machine interfaces has sparked interest in composite materials with flexible and sensing qualities. Iron (III) chloride (FeCl_3_) oxidant was utilized by Han et al. [264] to develop polypyrrole-coated CNC and CNF, which were then employed to strengthen polyvinyl alcohol (PVA). Self-healing nanocomposite films with superior mechanical strength (409% increase compared to pure PVA and high toughness up to 407.1%) and excellent adhesion (9670 times greater than its own weight) to various substrates in air and water were achieved by the combination of weak H-bonds and iron coordination bonds and the synergistic effect of these components. Liu et al. [265] synthesized a TOCN/Ti_3_C_2_T_X_ composite film using vacuum-assisted filtering and dispersion of 2,2,6,6-tetramethylpiperidinyl-1-oxyl (TEMPO)-oxidized CNF (TOCN). Good mechanical qualities are evident in the generated composite film that mimics the nacre-like lamellar structure of real shells. Additionally, the composite material has shown outstanding biocompatibility, antimicrobial, and electromagnetic shielding performance (36 dB). The piezoresistive sensor made from the composite film additionally displayed great sensitivity (11.6 kPa^−1^), quick response and recovery time (≤10 ms), an extremely low monitoring limit (0.2 Pa), and long-term stability (>10,000 cycles). It was also capable of picking up on human daily movements like chewing and finger bending.

For material-inherent uses, nanocellulose is interesting for photonic applications. The first is that CNCs have a liquid crystalline behaviour that produces opalescent films with a specific optical character; the second is that both CNF and CNC may generate optically clear, independent films. The adaptability of such materials lies in the structure and surface chemistry of cellulose. Nanocellulose can be easily made compatible with both hydrophobic and hydrophilic materials, employed as a host for optically active nanoparticles, and changed to accommodate covalently bound molecules [213]. For instance, CNF films are significant as substrates for optoelectronics as well as for applications like coatings and packaging due to their superior mechanical qualities and optical clarity.

Due to the enticing characteristics of chiral nematic photonic structures, CNCs have garnered significant attention for their applicability in optical applications. Simple evaporation of aqueous suspensions allows CNC to produce colored, nematic, iridescent, and chiral films. Hanif et al. [266] developed a water-stable flexible nanocellulose chiral nematic films through acid vapor cross-linked glutaraldehyde for chiral nematic templating. They reported that chiral nematic organization of the GA cross-linked CNC films was very well organized. Utilizing GA cross-linked CNC films as freestanding template substrates for conducting polymers (polypyrrole) and metal oxides (iron oxide) to construct flexible chiral nematic photonic hybrids increased the water stability of the films. In another study, chiral nematic and layered pseudonematic structural transitions result in reversible optical tunability in chiral photonic CNC) and elastic PU composite films. The composite films have remarkable mechanical flexibility and water resistance. During mechanical stretching and water absorption, the optical quality of the composite CNC/PU film can be modified reversibly. When a film stretches, the CNC structure changes from chiral nematic to layered pseudonematic. When the fixation is complete, shape recovery occurs when the fixation is exposed to water, and the CNC structure returns to its original chiral nematic order. The research and development of smart optical and mechanical sensors is furthered by these reversibly switchable form and optical qualities. Additionally, the aligned PLA/CNF nanocomposite film created by Geng et al. [267] is very transparent and exhibits anisotropic light scattering, which highlights its enormous potential for optical applications.

**Figure 12:**
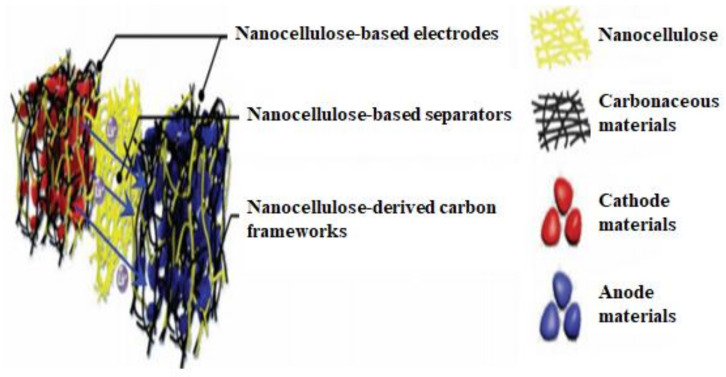
A conceptual illustration depicting of nanocellulose application in lithium–sulphur battery. Reproduce from ref. [136].

Nowadays, there is a growing focus on the use of nanomaterials in lithium-ion batteries. Nanomaterials have been widely used in lithium-ion batteries in several electronic gadgets such as smart mobile phones, notebook computers, and digital cameras. For an example, Hou et al. [268] synthesized the silicon/carbon composite nanosheets from waste vermiculite as high-performance anode materials for lithium-ion batteries. Interestingly, the developed anode shows the excellent specific capacity of ~948 mAh g^−1^ after 350 cycles at 1.0 A g^−1^ with a capacity retention of 85%. Previously, the use of current rechargeable lithium-ion batteries had several disadvantages such as insufficient power capabilities and suffer from serious safety risks due to their use of flammable liquid electrolytes [136]. Therefore, improvement has been made by developing the lithium–sulphur batteries as a promising energy storage device with high energy density and low cost. Traditionally, graphite has been used in these batteries as an anode electrode mainly because of its high electrical conductivity (~103 S/m) and theoretical capacity (372 mAh g^−1^) [269]. A big disadvantage of graphite-based electrodes is that they do not have sufficient energy density required to meet today’s energy demands and have a higher cost. The use of nanocellulose in lithium-sulphur batteries also has been widely discovered. Figure 12 shows a conceptual illustration of nanocellulose application in lithium–sulphur batteries. Several research papers in this area have shown good promise to begin a new era of long-lasting and high-energy batteries for a variety of applications such as in the military, transportation, solar panels, windmills as well as in space applications.

According to Nair et al. [270], the use of nanocellulose in gel polymer electrolyte resulted in excellent thermal stability (4200 °C), excellent conductivity (41.2 mS cm^−1^), and has a stable interface toward lithium metal. It also exhibited a specific capacity of ≈100 mAh g^−1^. Authors also found that the internal components of the cell were folded without any mechanical break. Therefore, it shows the potential usefulness of nanocellulose as an alternative electrode binder and the feasibility of lithium–sulphur batteries as a new power source. Interestingly, the developed nanocellulose-based gel polymer electrolyte had a stable capacity retention of up to 70 cycles. These stable cycling profiles show that the nanocellulose is an encouraging way toward the realization of green and reliable next-generation high energy lithium–sulphur batteries.

Besides that, Gou et al. [271] also developed nanocellulose as a cation in gel polymer electrolyte for lithium-ion batteries. The nanocellulose membrane with a porous network is prepared and is cross-linked with glutaraldehyde to further its application as a gel polymer electrolyte, and the skeleton membrane that is cross-linked by 6 wt.% glutaraldehyde exhibits satisfactory mechanical properties and thermal stability. Moreover, the gel polymer electrolyte has moderate electrochemical performance and reverse capability.

## 7. Future Prospects and Challenges

The demand for nanocellulose materials is increasing substantially due to their desirable properties such as high tensile strength, large aspect ratio, low density, and high degree of thermal stability. They are also derived from renewable biological sources and are biodegradable. As discussed in detail in the preceding section, nanocellulose is widely used, frequently on a large scale, in pulp and paper production, the packaging industry, energy-based applications and environmental remediation.

It is also increasingly used, albeit on a smaller scale, in the electronics, food, construction and cosmetics sectors. The nanocellulose market was worth to USD 346 million in 2021 and is projected to increase USD 963 million by 2026, growing at a CAGR of 22.7%. There are rising concerns regarding the environment along with the market push for greater sustainability in the development of end-products, demanding the utilization of nanocellulose. The rising resource constraint is also shifting the demand toward biomaterial products, which is a significant expansion of the nanocellulose market [272].

The rapid expansion of the nanocellulose market is driven by their broad applicability and unique properties. From that, CNF accounted for the largest share of the nanocellulose market in 2018, followed by CNC. There are several commercial entities that produce nanocellulose at industrial scale, of which the most prominent are CelluForce Inc., (Montreal, QC, Canada), Innventia (Stockholm, Sweden), InnoTech Alberta (Edmonton, AL, Canada), American Process Inc. (Atlanta, GA, USA), Blue Goose Biorefineries Inc. (Saskatoon, SK, Canada), FPInnovations (Pointe-Claire, QC, Canada), Chuetsu Pulp & Paper Co. Ltd. (Takaoka, Japan), Dai-ichiKyogo (DKS) (Tokyo, Japan), Borregaard (Sarpsborg, Norway), Nippon Paper Group (Tokyo, Japan), CelluComp Ltd. (Burntisland, UK) and VTT Technical Research Centre (Espoo, Finland). It is difficult to provide a clear estimation of the end-use product cost due to the confidentiality of the financial-related aspects of commercial entities. In 2017, one can purchase CNCs with a wide range of structural properties and surface chemistry for a price ranging from USD 4 to USD 10 per kilogram [273]. Nevertheless, considering the price of the raw material (around USD 0.5 per kilogram) and production cost, we expect that cost of CNFs will range between USD 7 and USD 12 per kilogram of dried material.

Nanocellulose plays a crucial role in improving the mechanical and barrier properties of final products as already described. The current nanocellulose markets are showing rapid growing rates, particularly in Japan and Canada, with a variety of products already available in the field of consumer goods, textiles, cosmetics and sanitary products. The demands of these commodities are widely increasing due to its high strength, stiffness and light weight. The foremost vendors of nanocellulose are CelluForce (Canada), Nippon (Japan), American process Inc. (US), Cellucomp (UK), Blue Goose Refineries (Canada), VTT (Finland) and so on. In Malaysia, ZoepNano Sdn. Bhd. is the pioneer in nanocellulose commercialization, where they use palm-based biomass to produce their nanocellulose. All these companies are continuously improving their shares in the market by adopting newer strategies and agreements in order to achieve versatile demands for their products in the global markets. The nanocrystalline cellulose market is expected to further expand as the technology advances and equipment used to manufacture them becomes cheaper. The produced CNFs are mainly utilized as resins, fillers, or thickeners for composites production. However, CNCs find applications in diverse markets like textiles, paints, personal care, construction and electronics [272].

## 8. Economic Assessment of the Nanocellulose Extraction Process

Several methods have been proposed for the extraction of nanocellulose as discussed in the previous section. These processes remove the amorphous cellulose either partially to obtain CNF or completely to obtain CNC. Currently, concentrated mineral acids like sulfuric acid are predominantly used in hydrolysis for the industrial production of CNC. However, there are significant downsides with using sulfuric acid, including reduced thermal stability of the CNC as compared to CNC produced via other methods and its difficulty in being functionalized, owing to the sulphate groups that reside at the surface of the biopolymer. Additionally, the process for recovering the acid is laborious [274,275]. Assis et al. [276] mentioned that conventional sulfuric acid hydrolysis for CNC production necessitates acid-resistant equipment, increasing the capital expenditure (CAPEX). The CAPEX is further increased by acid recovery through neutralization and effluent treatment. According to a study on CNC, the cost of manufacturing sulfuric acid hydrolysis ranged from USD 3632 to USD 4420 per dry ton.

Research studies have looked into the idea of employing different mineral or organic acids as an alternative to the conventional sulfuric acid approach to producing CNC. Techno-economic analysis (TEA) by Bondancia et al. [277] revealed that the cost of the citric acid applied in the hydrolysis had a significant effect on the minimum selling price (MSP) of CNC, which was relatively higher at USD 16,460 per dry ton. The capital cost of CNC production via citric acid hydrolysis was USD 7,000,000, which was comparatively lower. On the other hand, operating expenses were higher at USD 8.25 million, with citric acid alone accounting for 40% of this cost even with an acid recovery rate of 89%. It was predicted that the MSP of CNC produced using citric acid and sulfuric acid would be similar for a 79% decrease in the market price of citric acid, or at USD 290 per ton. There is a trade-off between the economic and environmental aspects of producing nanocellulose, as can be evidenced looking at the MSP with a chemical recovery section and citric acid hydrolysis, suggesting that the production of CNC using citric acid could be explored in the context of a biorefinery concept, providing a sustainable and ecologically friendly way to acquire a valuable nanocellulose. In another study, CNC were extracted from sugar cane bagasse using sulfuric acid (S-CNC), citric acid (Cit-CNC), and their combination (Cit-S-CNC). They stated that the high cost of citric acid recovery was a critical contributor in the minimum product selling price values established by TEA for Cit-CNC and Cit-S-CNC, being 34.0% and 37.2% higher, respectively, than S-CNC. The hydrolysis and recovery of citric acid had a significant adverse effect on the environment, contributing up to 58% of the global warming possibilities for Cit-CNC and Cit-S-CNC, which were more than twofold higher than for S-CNC [278].

Norrrahim et al. [13] treated the oil palm mesocarp fiber (OPMF) with superheated steam and potassium hydroxide pretreatments, followed by mechanical fibrillation utilizing wet disc milling. They reported that operating cost of producing CNF by superheated steam-wet disc milling is estimated at USD 55.6/kg, which is much higher than the operating cost of producing potassium hydroxide-wet disc milling at USD 48.9/kg. Superheated steam and potassium hydroxide pretreatment are expected to generate gross profits of USD 103.3 thousand and USD 109.9 thousand, respectively, in a year of operation at the established selling price of USD 240.62. Moreover, positive net present values and high internal rates of return demonstrate that both pretreatments are profitable and anticipated to produce adequate revenue growth.

Alternatively, enzyme-mediated production of nanocellulose can potentially provide a way to satisfactorily address both the environmental and economic constraints. It should be emphasized, though, that commercial enzymes are frequently tailored to convert cellulose and hemicellulose fragments into fermentable sugars. In order to enhance the economic viability of these conflicts, more research is required on improving enzyme combinations for the production of nanocellulose and on-site enzyme production [279,280].

Nevertheless, industrial extraction of nanocellulose has significant obstacles, including the need for low-cost raw materials, environmentally friendly processing, higher operating costs, and extraction techniques that are rapid and cost-effective [281]. According to Jiang et al. [282], most investigations on the production of nanocellulose used purified or bleached chemical pulp that had already lost 50 to 60% of its biomass content. Owing to lower yields of nanocellulose compared to the original biomass, this approach causes a less effective exploitation of the biomass. In contrast, mechanical pulp can be a viable alternative, but it requires deeper research. Additionally, extensive research should be focused on improving the reaction yield, which will enhance the total economics of the production route [283].

## 9. Conclusions

Due to the urgent need for sustainability and environmental preservation, there is a steadily growing interest in developing biodegradable and renewable materials. Nanocellulose is an environmentally friendly material for advanced technologies in creating various applications that are becoming increasingly vital. The uses of nanocellulose in numerous fields are attractive because of its renewable, biocompatible, and biodegradable nature. For sustainable applications, nanocellulose in diversified areas include packaging products, biomedical, electronics, automobile body parts/interiors, constructions, textiles, paper making, water purification, and sensors. The primary function of designing is to establish the possibilities, drawbacks and acceptability of nanocellulose for sustainable applications. The development of sustainable applications depends on end-to-end issues such as raw material selection, extraction methods, product design and life cycle. This review elaborates on the importance of nanocellulose as a valuable natural resource and many vital aspects associated with its invention. This includes processing parameters involved in its extraction from cellulose feedstock, the structural and morphological characteristics of nanocellulose before and after extraction, and surface modifications to ensure greater applicability of nanocellulose in targeted applications. The application of nanocellulose in packaging, paper, energy and electronics, and bio-medical and environmental remediation fields are also encompassed.

As discussed, the isolation processes that involve the pre-treatment process play a decisive role in dewaxing, removing the lignin and other impurities embedded in the cellulose fibers and improving the quality of nanostructured materials (cellulose microcrystals and cellulose microfibrils), CNF and CNC. However, BC depends on their morphologies, origin and isolation techniques that originally produced a concentrated cellulose, which is the degree of crystallinity index followed by CNC and CNF, accordingly. The method used to extract CNCs and CNFs significantly affects their structural and physical-chemical properties. Surface modification techniques, including chemical modification and adsorption of surfactants, are used to improve the dispersibility of CNCs and CNFs within the hydrophobic and hydrophilic polymer matrix. The continued research in this field shows that reliable and reproducible processes are essential to ensure their uptake by industry. Current production processes for CNCs and CNFs involve multiple steps, requiring strong chemicals and harsh reaction conditions. Future studies are expected to develop facile and single-step extraction processes involving mild reaction conditions where preventive measures are also needed to reduce structural damage during acid hydrolysis. The damage to the cellulose during acid hydrolysis can be reduced by introducing pre-treatment processes; however, multiple steps are considered costly and restrict their greater commercial exploitation. Hence, preserving and improving the nanocellulose morphology and structure with a simple, efficient design process and cost-effective processing should be at the forefront of coming research. The promise of nanocellulose to be incorporated as a filler component in a polymer matrix to develop biodegradable green nanocomposites is one of the most significant steps towards environmental sustainability. Besides that, there is a need for more laboratory-scale research on the use of nanocellulose for sustainable applications, with the group effort of two or more areas of expertise, like scientists, professionals, engineers and designers. Commercial manufacturing of nanocellulose adapted to different end-user applications would have a promising future in expanding global technology development, with strong collaboration between industries, researchers and academicians.

In terms of nanocellulose toxicity, when investigated under realistic doses and exposure scenarios, nanocellulose has a limited associated toxic potential, albeit certain forms of nanocellulose can be associated with more dangerous biological behaviour due to their specific physical characteristics. Further, several studies on cellulose–human interactions confirmed the toxicity potential of nanocellulose in terms of cytotoxicity in various experimental systems, especially during isolation. According to the data highlighting a potential risk associated with nanocellulose, these possibilities can be avoided or reduced by avoiding those nanocellulose types with extreme length more than 5 m, overload doses, or in a physical format that causes adverse biological effects, like freeze-dried and re-suspended powder. The lack of knowledge about the incidence, in-situ exposure doses, and the particular types of nanocellulose most frequently used appears to be the limiting factor in guiding the scientific output regarding nanocellulose toxicity; therefore, commercial products should be tested rather than in-house productions. Furthermore, a clear understanding of the physical and chemical properties of currently produced and used nanocellulose and realistic exposure doses is of the utmost importance, and it is inevitable according to the life cycle of nanocellulose-based composite materials from five different stages: the production of raw materials or isolation (Stage 1), manufacturing (Stage 2), transportation (Stage 3), consumer use (Stage 4) and disposal (Stage 5).

## Figures and Tables

**Table 1:** The physical comparison between nanocellulose.

Nanocellulose Type	Degree of Polymerization	Crystallinity/Crystal Structure
Bacterial nanocellulose (BNC)	4000–10,000	Iα (shell) and Iβ (core)—the highest degree of crystallinity
Cellulose nanofiber (CNF)	≥500	Primarily Iβ—lowest degree of crystallinity
Cellulose nanocrystals (CNC)	500–15,000	Primarily Iβ, sometimes Iα—medium degree of crystallinity

**Table 2:** The dimension of nanocellulose is produced from different plant fibers.

Source	Type of Nanocellulose	Diameter (nm)	Ref.
Kenaf	CNC	45–55	[64]
Miscanthus × giganteus	CNF	10–20	[65]
Flax	CNC	20–100	[66]
Hemp	CNC	20–100	[66]
Jute	CNC	16	[67]
Sisal	CNF	27	[68]
Sugar palm fiber	CNC	8.5	[69]
Banana	CNC	14.02	[70]
Abaca	CNF	3.12	[71]
Bamboo	BCNF	15.02	[72]
Sugarcane bagasse	CNF	20–30	[73]
Coconut fiber	CNC	13.7	[74]
Pineapple leaf fiber	CNC	68	[75]
Wheat straw fiber	CNC	10–80	[76]
Kenaf bast	CNC	10–60	[49]
Kenaf bast	CNF	2–6	[77]
Wood	CNF	15	[78]
Bagasse	CNF	5–15	[79]
Soy hulls	CNF	20–120	[80]
Rice straw	CNF	4–13	
Soybean stock	CNF	50–100	[79]
Cotton	CNF	10–25	
Oil palm empty fruit bunch	CNF	18	[53]

**Table 3:** The yield of nanocellulose obtained per 100 kg of raw material from different sources.

Source	Type of Nanocellulose	Yield (kg)	Ref.
Sugarcane bagasse (SB)	CNC	11.3	[81]
Sugarcane straw (SS)	CNC	12	[81]
Wheat straw	CNF	42.3	[82]
Banana peel	CNF	27–71	[83]
Wood flour	CNC	11.43	[84]
Grapevine stems	CNF	15–20	[85]
Grapevine pomace	CNC	10–15	[85]
Cotton linters	CNC	80	[86]

**Table 4:** List of various physical approach of functionalization of nanocellulose.

Treatment Type	Definition and Requirement	Benefits	Significant Findings	Ref.
Plasma	An electric discharge that produces partially ionized gas under vacuum at room temperature Most common and traditional plasma technique, known as “cold” plasma (or non-thermal), the temperature in a non-thermal plasma surface treatment typically ranges from 20°C to 30°C, allowing enough gas ionization to functionalize organic substrates	Cost-effective and efficient physical surface treatment method for nano cellulosic materials Selective modification to improve surface compatibility while keeping the bulk properties of materials intact. Improve the bonding and functionality of the reinforcement and matrix surface properties Low-cost and simple technique for modifying polymer surfaces for a variety of applications, including improved bonding in polymer-matrix composites	The treatment of nanocellulose with zinc oxide (ZnO) plasma completely inhibits the growth of Staphylococcus aureus, later has been proposed as an environmentally friendly solution for the food packaging industry Improve surface modification of bacterial nanocellulose, resulting in morphology structure shifting and associated properties such as decreased water flux and active pore area	[139,140]
Flame	A high-speed process used to improve the adhesion of materials Applying a gas flame to a material’s surface to increase adherence The free radical degradation mechanism underpins flame treatment. Heat, oxygen atoms, and hydroxide radicals are the three main ingredients needed to produce polymer radicals. Polar functional groups are added, and molecular chains are disrupted by rapidly applying high temperatures to a surface.	By using this method, a large surface might be covered. Controlling the flame chemistry, the distance between the surface and the flame, and the passage of time can produce good results by using this technique Flame treatment is significantly quicker than corona treatment, but it necessitates more complex and costly equipment.	To improve the binding between polyethylene, a flame treatment was applied to a paperboard coated with a nanocellulose film	[141]
Corona	Low-temperature visible electrical discharge that modifies the surface’s qualities.	Increases the material’s surface energy, which boosts bonding with various coatings and adhesives but simultaneously lowers the electrical conductivity. Enhances a matrix-fiber (polymer/cellulose fiber) system’s adhesion.	The mechanical properties of a cellulose-polypropylene composite improved after corona modification of the fiber. Corona treatment of the fiber alone is sufficient to significantly improve the physicomechanical properties of the composite material. When both components are modified under corona discharge, the results are slightly better than when only one component is treated fiber. A rise in yield stress was also observed for poly(lactic acid) composites containing corona-treated miscanthus fiber.	[142]
Laser	One of the most accurate techniques for small-scale surface alterations. During the procedure, the substance interacts with laser radiation in a way that may cause melting, direct vaporization, or low-temperature plasma.	A functionalized paper based on gold nanocellulose particles (Au NPs) conjugated graphene oxide (GO) was developed for photothermal ablation against infectious bacterial pathogens using a near-infrared (NIR) laser, exhibited a significant improvement in tensile strength, bursting index, and tear index when compared to pure nanocellulose paper. Improve dimensional stability.	Laser treatment of bacterial nanocellulose material resulted in some structural changes but no chemical change.	[143]
Hydrothermal	Nanocellulose acts as the reducing agent and stabilizer Includes modifying and partially degrading lignocellulose biomass components (in a slurry) using water heated to between 150 °C and 230 °C.	Reaction time takes long time and requires high concentration of NaOH	Hydrothermal treatment using buffered media at different temperatures enhanced the dimensional stability of the treated samples After hydrothermal treatment of cellulose nanocrystals, 1–2 nm in height with micrometer range length fiber structures of carbon nanotubes (CNT) was found under AFM	[144,145]
Ion Beam	Precise control of implantation fluency, profile, temperature, and dopant/ion species. Effective methods for characterizing and modifying materials by blasting solids with intense ions.	Used to introduce impurities into matrices by ion implantation, to produce nanocomposite materials by ion beam deposition, to create nano-patterned surfaces of materials by ion beam erosion, or to research unique properties resulting from impurity-defect interactions.	A novel method of cellulose breakdown could be obtained by the application of low-energy N+ processed cellulose Results in the creation of high-performance polymers for use in various optical components and luminous devices. Target sputtering rate and ion current density are two characteristics that can be controlled by the method, which is not possible with evaporative approaches or magnetron sputtering. Precision coatings can be produced by controlling these factors separately, which is suitable for industries like semiconductor fabrication or precision optics where high-quality films are essential.	[146,147]

**Table 5:** Comparison of various chemical approach of functionalization of nanocellulose.

Treatment Type	Definition and Requirement	Benefits	Significant Findings	Ref.
Alkalization/Mercerization	Chemical treatment uses different concentrations of sodium hydroxide to remove impurities from the fiber in order to increase compatibility during internal reinforcement and thermoplastics. Well-known technique for surface treatment because it is effective in lowering the moisture content of fibers. Moisture-resistant nanocellulose composites are made possible by a particular process containing an alkyl functional group.	Fiber bundles disintegrate (swell), producing smaller, better-quality fibers and better fiber wettings. Alkalized cellulose nanofibers have greater thermal stability than unalkalized cellulose nanofibers. The alkyl groups have taken the place of the hydroxide groups in this. Alkalization reduces the moisture sorption of the treated nanofibers.	It was discovered that using ethanol as a dispersing medium and adding alkaline nanoparticles works synergistically to increase interactions between grafted cellulose nanocrystals, resulting in cluster formation. The treatment of nanocellulose from rice husk (with NaOH) resulted in the complete removal of the outer surface, and all results appear to indicate that the final sub-product could be cellulose II devoid of hemicellulose, waxes, or silica.	[149]
Silylation	The most commonly used organosilanes undergo hydrolysis, condensation, bonding with OH groups, and formation of covalent linkages. Silane modifiers are typically used in aqueous and alcoholic solutions, as well as aqueous emulsions.	By using this method, it was discovered to improve the nanocellulose/polymer interface and provide additional reinforcing properties. It was also discovered that silane-treated nanocellulose fibers improved the physicochemical properties of composite materials. Reaction with hydroxyl groups in nanocellulose fibers, improving surface and mechanical properties.	Loading the matrices with silane-treated cellulose short fibers improved the tensile strength and modulus of the composites when compared to non-treated counterparts.	[150,151]
Grafting	Grafting of different monomers onto the surface of cellulosic fibers, triggered by free cellulose radicals.	Grafting chemicals reduce water absorption rate via reaction with fiber hydroxyl groups, resulting in increased mechanical strength. Vinyl monomers can improve nanocomposites’ termite and fungal resistance, as well as their flame retardancy. Acrylonitrile grafting improves thermal stability.	The material properties of nanocellulose/polymer composites improved by adjusting the miscibility of surface-grafted layers on nanocellulose in the polymer matrix. Grafted cellulose nanocrystals (CNCs) are effective at flocculating kaolin (a model wastewater treatment system), the freshwater microalgae *Chlorella vulgaris*, and the marine microalgae *Nannochloropsis 21culate*.	[152,153,154]
Etherification	One of the most-often-used methods of modification that is used to cationize the surface of nanocellulose, a highly alkaline reaction medium with compatible organic epoxide is widely used. Functional chemicals, such as ethers, react with cellulose hydroxyl groups and cross-link the cellulose interchain, resulting in reduced water uptake and dimensional stability.	Improvements in the cell wall’s stability, which reduces water absorption and increases resistance to fungus attack, as well as dimensional stability and thermal stability of fibers. Increases in mechanical characteristics, chemical resistance, and moisture regain that is substantial.	The observed inhibitory impact is increased when the covalent connections are resistant to enzymatic hydrolysis, as they are with etherified CNF. Improved dispersibility of the starch and nanocellulose composite material.	[155]
Sulfonation	A method for coating the surface of nanocellulose materials with anionic charges. The hydrolysis of the sources is catalyzed by concentrated sulfuric acid employed in CNC synthesis, which also facilitates the generation of sulphate half-esters from CNC hydroxyl groups.	Due to a lower density of surface sulfate groups, these spherical CNCs demonstrated better thermal stability compared to those obtained by hydrolyzing with pure sulfuric acid. Sulfonation is a green technique because it does not require halogenated wastes.	Effective dispersive micro solid-phase extraction and detection of silver nanoparticles in food products using sulfonated nanocellulose. Development of nanocellulose/sulfonated carbon nanotube hydrogel films, a super-tough and ultra-sensitive flexible electronic skin is created. Sulfonated multi-walled carbon nanotubes (SCNTs), had a very high sensitivity of roughly 4.4 kPa^−1^, an incredibly quick response time of about 10 ms, an incredibly low detection limit of 0.5 Pa, good stability (>11,000 cycles), and a mechanical strength of up to 184 MPa.	[156,157]

**Table 6:** Advantages and disadvantages of each processing method for nanocomposite fabrication.

Fabrication Method	Advantages	Disadvantages
Solution casting	Provide better uniformity of thickness and clarity than extrusion. The nanocomposites have a fine surface and better physical properties and flexibility.	The polymer needs to dissolve in either water or a volatile solvent. It is important to generate a stable solution with a suitable minimal solid content and viscosity.
Melt intercalation	Due to its simplicity, it is frequently utilized to fabricate polymer nanocomposites. Ideally suited to a variety of industrial processes, including extrusion and injection molding, so that it can be commercialized. Low cost-effectiveness and eco-friendly (solvent free)	Getting the nanocellulose to disperse properly in the polymer matrix can be more challenging compared to other approaches. The usage of high temperatures could damage the nanocellulose properties. Not ideal to temperature sensitive polymers
Impregnation	Faster and inexpensive fabrication method. Allows the final properties to be controllable in advance.	Needs low viscous polymers. Difficult to obtain even dispersion of nanocellulose on the surface.
In-situ polymerization	Controllable nanocellulose morphology. Have covalent bonds between the polymer chains and functional groups in nanocellulose, and both thermoset and thermoplastic polymers can be used. Ideal for polymers that are susceptible to temperature and solution.	Ease of agglomeration and required extra curing chemicals. Must comply with the requirements for polymerization.
Coating	Simple technique. Low investment costs. High availability. Possibility of automation.	High consumption of coating materials. Difficulty to control the thickness of coating layers.

**Table 7:** Key applications and potential advantages of nanocellulose.

Application Area	Properties	Key Application
Food packaging	Flexible, rigid, improved barrier	Packaging films
Biomedical	Nontoxic, excellent biocompatibility and biodegradability	Scaffolds, water absorbent pads, antimicrobial films and tampons, sanitary napkins or wound dressing
Cosmetics	Durability, compatibility, good elasticity	Composite coating agent for nails, hair, or eyelashes
Electronics	High dielectric, excellent mechanical and biocompatible	Sensor, electronic displays and windows
Optical materials	Crystalline, flexibility, biocompatible	Electronic transistor, sensors
Automobile	Good electrical, thermal, magnetic, physico-chemical properties	Lightweight and high strength components such as bumpers, side panels and dashboards
Constructions	Increase fracture toughness, cheaper, low density, high strength	Blocks, sensors to monitor stress levels in bridge
Aerospace	High strength, light weight	Windows, sensors
Textiles	Easy care, low impurity, good mechanical strength, biocompatible	Antimicrobial medical field, paste printing
Paper industry	Easy availability, eco-friendly, renewability	Grease-proof paper
Water purification	Biosorable, low cost, nontoxic	Filtration

**Table 8:** Recent developments in nanocellulose-based composites for sensing applications.

Sensor Type	Target	Composite Description	Detection Limit	Ref.
Gas sensor	Ammonia	QCM/CA/PEI/ GO	1 ppm	[236]
		Cellulose/TiO_2_/PANI	2 ppm	[237]
	Nitrogen dioxide (NO_2_)	CNC/Fe_2_O_3_	2 ppm	[238]
Chemical sensor	Formaldehyde	PEI/BC/QCM	1 ppm	[239]
	Catechol	CB/CNC/NR	-	[240]
	Toluene	rGO/CNC/NR	-	[241]
Enzyme sensor	HNE	Peptide/CNC	50 mU mL^−1^	[242]
	Cellulase	XG/CNC	-	[243]
	Xylanase	CX/CNC	-	[243]
	Trypsin	Peptide/PVA/CNC	20 µg mL^−1^	[244]
Ion sensor	Fe^3+^	Py/CNC	10^−3^ × 10^−3^ M	[245]
	Pb^2+^	CNINH/CNC	7 × 10^−11^ × 10^−3^ M	[246]
	Cu^2+^	CPC/CNF	0.2 × 10^−6^ M	[247]
Glucose sensor	Glucose	GO/CNF	250 mg L^−1^	[248]
		Ag/CNC	0.116 × 10^−6^ M	[249]

Abbreviations: QCM: quartz crystal microbalance; CA: cellulose acetate nanofibers; PEI: polyethyleneimine; GO: graphene oxide; polyaniline (PANI); iron oxide (Fe_2_O_3_); CB: carbon black; NR: natural rubber; XG: xyloglucan; CX: cationic xylan; Py: pyrene; CNINH: iron sensitive fluorescent ionophore N′-(4-cyanobenzylidene) isonicotinohydrazide; CPC: cyanobacterial C-phycocyanin.

## Data Availability

Not applicable.
